# Tracing mobility patterns through the 6th-5th millennia BC in the Carpathian Basin with strontium and oxygen stable isotope analyses

**DOI:** 10.1371/journal.pone.0242745

**Published:** 2020-12-09

**Authors:** Margaux L. C. Depaermentier, Michael Kempf, Eszter Bánffy, Kurt W. Alt

**Affiliations:** 1 Department of Early Medieval and Roman Provincial Archaeology, University of Basel, Basel, Switzerland; 2 Department of Archaeology and Museology, Masaryk University, Brno, Czech Republic; 3 Institute of Environmental Social Science and Geography, University of Freiburg, Freiburg, Germany; 4 German Archaeological Institute, Roman Germanic Commission, Frankfurt a. M., Germany; 5 Center of Natural and Cultural Human History, Danube Private University, Krems-Stein, Austria; 6 Integrative Prehistory and Archaeological Science, University of Basel, Basel, Switzerland; University at Buffalo - The State University of New York, UNITED STATES

## Abstract

The complexity of Neolithic population movements and their interpretation through material culture have been the subject of archaeological research for decades. One of the dominant narratives proposes that groups from the Starčevo-Körös-Criş complex spread from the central towards the northern Balkans in the Early Neolithic and eventually brought the Neolithic lifestyle into present-day Hungary. Broad geographical migrations were considered to shape the continuous expansion of Neolithic groups and individuals. However, recent archaeological research, aDNA, and isotope analyses challenged the synchronous appearance of specific material culture distributions and human movement dynamics through emphasizing communication networks and socio-cultural transformation processes. This paper seeks to retrace the complexity of Neolithic mobility patterns across Hungary by means of strontium and oxygen stable isotope analyses, which were performed on a total of 718 human dental enamel samples from 55 Neolithic sites spanning the period from the Starčevo to the Balaton-Lasinja culture in Transdanubia and from the Körös to the Tiszapolgár cultural groups on the Great Hungarian Plain (Alföld). This study presents the largest strontium and oxygen isotope sample size for the Neolithic Carpathian Basin and discusses human mobility patterns on various geographical scales and throughout archaeological cultures, chronological periods, and sex and gender categories in a multiproxy analysis. Based on our results, we discuss the main stages of the Neolithisation processes and particularly trace individual movement behaviour such as exogamy patterns within extensive social networks. Furthermore, this paper presents an innovative differentiation between mobility patterns on small, micro-regional, and supra-regional scales, which provides new insights into the complex organisation of Neolithic communities.

## Introduction

Population movements on different geographical, temporal, and demographic scales during the Neolithic have since long been posited through the interpretation of the archaeological material culture. One of the dominant narratives proposes that groups from the Starčevo-Körös-Criş complex, which spread from the central towards the northern Balkans in the Early Neolithic, moved farther north and brought sedentism to present-day Hungary [1–4]. According to recent archaeological and molecular genetic data, the western Carpathian Basin (Transdanubia) is also the cradle of the first farmers of Central Europe, represented by the Linearbandkeramik (LBK) culture [5–7]. The springboard for this study was a German-Hungarian interdisciplinary research project that aimed to investigate the settlement and population history of the Carpathian Basin in the Neolithic (and Chalcolithic) and their influence on the Neolithic settlement of Central Europe.

By integrating scientific methods such as anthropology, molecular genetics and biogeochemistry, and comparing them with the chronological sequence and archaeological material types in the project, important aspects of the nature and dynamics of settlement and population development in the Carpathian Basin could be depicted in their temporal depth over the entire Neolithic and the first half of the Chalcolithic. Within the framework of population genetic analyses, we divided the Carpathian Basin into two main regions: Transdanubia and the Great Hungarian Plain (Alföld). The archaeological and demographic data required for the evaluation of the sites were provided by staff members of the Institute of Archaeology of the Hungarian Academy of Sciences and by many other Hungarian archaeologists and anthropologists. The molecular genetic data and conclusions of two dissertations written by Anna Szécsényi-Nagy and Victoria Keerl have in part been already published [6–8] and are now continued in parallel at the full genome level [9–11]. This paper focuses on another part of this project and presents the results of extensive strontium and oxygen stable isotope analyses.

The determination of strontium isotope baselines for this research area, which are based on the interpretation of faunal dental enamel, human bone and shell strontium isotope data, and on their integration with multivariate environmental analyses, have been published in a separate paper [12]. In each case and because of the often small baseline sample size, we integrated multivariate environmental analyses and suggested a distinction between “site-specific” and “micro-regional” baseline ranges [12]. Strontium and oxygen stable isotope analyses enabled the reconstruction of mobility patterns on various temporal and spatial scales through the 6^th^–5^th^ millennia BC in Transdanubia and in the Alföld. The main questions are as follows: Are there any disparities in mobility patterns between geographical areas, archaeological cultures, chronological periods, and/or between sex and age categories? What kind of mobility patterns can be identified for the Neolithic period? How can the results of isotope analyses be compared to the archaeological records and the results of aDNA-analyses in this research area?

### Archaeological background

The shift to sedentary life and food production is certainly one of the major developments in human history. The Carpathian Basin appears to have been culturally divided into two main regions with somewhat different traditions, although both shared roots in the northern Balkans [2, 13]. The Körös presence east of the Tisza and, above all, on the eastern bank of the River Danube [3] and the Starčevo territory west of the Danube are the formations with the greatest relevance for the present study.

The Körös groups reached the heartland of the Carpathian Basin around 6000 cal BC: after crossing the River Maros, they gradually occupied the floodplain of the three branches of the Körös rivers on the southern Alföld plain, a region abounding in water, advancing from south to north [14, 15]. The Starčevo groups entered the Carpathian Basin from Serbia and northern Croatia. They crossed the Danube and the Drava and established their most intensive settlements on the alluvial plains of the rivers. Their settlement activity was very intensive in a small area in south-eastern Transdanubia; the long-lived Alsónyék settlement begins with the first farmers’ occupation, the so-called Starčevo period. One of the main areas for sampling and stable isotope investigations is located here, in the Sárköz region in south-eastern Transdanubia.

The long-lasting process of gradual change suggests that the genuine change in subsistence strategies did not occur in the initial phases of the western Transdanubian Neolithic, but one phase later, at the time when longhouses appeared, in the period hallmarked by the Transdanubian Linearbandkeramik (TLBK). This assumption is confirmed by the observation that the shift to food-production did take place, although not in the earliest TLBK phase, but some three or four generations later [2, 16]. Simultaneously, we witness the emergence of the earliest Alföld LBK (ALBK) in the east and the chain of early ALBK sites along the northern fringes of the Alföld led to the conclusion that the formation of the ALBK (i.e. the Neolithic transition in that area) occurred practically without the participation of groups other than the late Körös.

A contact area lies along the boundary between the lowland and the mountains, where strong genetic relations between the early Alföld Linear Pottery (ALP) and the Körös culture can be demonstrated [6]. Local farming groups that lived on the fringes of the Alföld most probably withdrew into the mountains and only in a later phase of the eastern Hungarian Neolithic did these groups begin to merge with the ALBK: their traces can be identified in the later local ALBK groups such as the Tiszadob, Szilmeg, Bükk, and Esztár groups of the developed ALBK [17, 18]. Some features of the material culture appear to be alien to the ALBK heritage, while certain new traits can also be found in southern groups such as the Szakálhát group, making their isolated highland origin somewhat dubious [19].

Thus, after the formation of the Alföld variant of the LBK group, rooted in the Körös and some indigenous traditions, the South-East European Neolithic world seems to have revived with the onset of the local Late Neolithic, with the spread of genuine Balkanic features borne by the Szakálhát and the Tisza populations in the earlier 5^th^ millennium BC. The Late Neolithic of the Tisza region was determined by the emergence of tell settlements surrounded by an extensive network of satellite settlements, whose significance is now somewhat better understood in the wake of extensive field prospections [20].

Local groups of the TLBK population appeared also during the later Transdanubian centuries. The earlier hypotheses about the temporal or at least the clear spatial distinctness of the Keszthely, Notenkopf and Zseliz phases have been discarded, and more recent research [21] has demonstrated a fairly complex and often overlapping network, based mainly on architecture, pottery styles and absolute chronological data. As for the Sárköz region and south-eastern Transdanubia in general, impulses arrived repeatedly from the northern Balkans, both in terms of the influx of groups such as the Sopot communities from northern Croatia and the early Vinča groups arriving mainly from the Voivodina [16, 22]. These southern impacts all shaped the late Transdanubian LBK [23]. Finally, the huge Lengyel cultural tradition (that extended from the Sárköz region to eastern Austria and Moravia in the west, and Slovakia and even Lesser Poland in the north) was imbued with South-East European impacts. Alsónyék, which had a dense TLBK occupation phase and a probably large Sopot settlement within eyesight of the Lengyel houses, is currently the largest and most important Lengyel site in Europe [24, 25]. With its over 120 sturdy timber-framed houses and no less than 2300 burials as well as an unparalleled population growth around 4700 BC, the site is an invaluable source for evaluating diet and mobility through stable isotope investigations. A detailed version of this case study has therefore been published in a separate paper [26].

In the aftermath, i.e. in the later 5^th^ millennium BC, at the time when the Vinča tradition disintegrated in the northern Balkans, this period saw yet another distinct influx, bringing southern features into the late Lengyel material culture. In southern Transdanubia, the impacts were decisive, and the new cultural formation was labelled the Balaton-Lasinja culture, which reflects surviving Lengyel traditions with Balkanic Chalcolithic features. These impacts are weaker in northern Transdanubia, while in south-western Slovakia, Lengyel features remained predominant. In the east, after the long life of the tell settlements, some changes can be detected in the mid-5^th^ millennium BC, marked by the proliferation of more short-lived settlements and the abandonment of most tells, alongside the appearance of large formal cemeteries separate from settlements [27]. The Tiszapolgár culture, flourishing at the onset of the Chalcolithic in the Alföld, still retained some characteristics of the previous tell settlement period of the Tisza-Herpály-Csőszhalom complex; one of the recent research questions is what these structural changes meant in terms of demographic issues, nutrition and lifestyles or mobility [28, 29].

### Material

**Fig 1:**
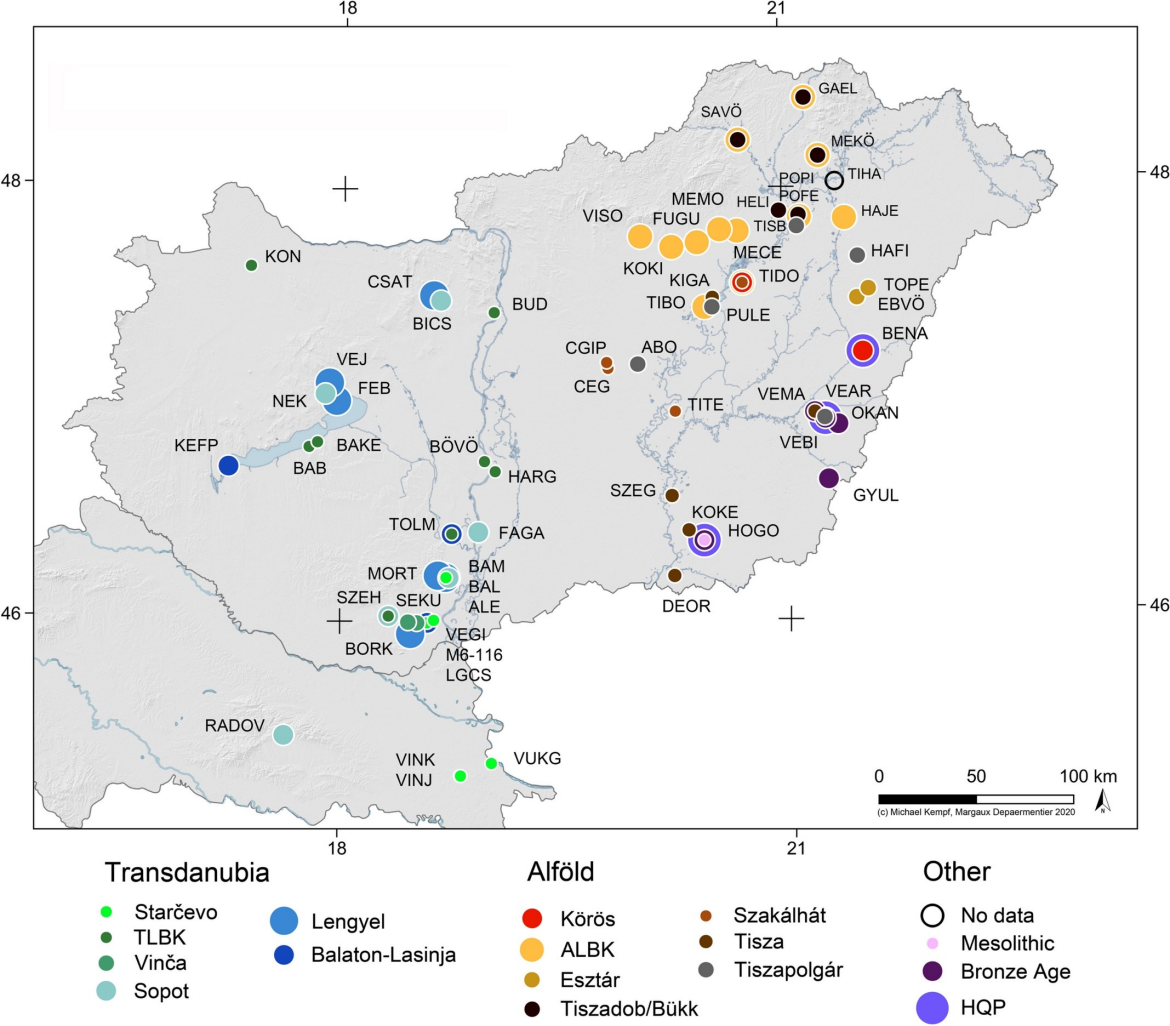
Distribution of the sites selected for human dental enamel samples for strontium isotope analyses. Some sites provided samples from different archaeological cultures that are marked by overlapping coloured dots. The full name of each site can be found in the supporting information to this article (**Table A in S1 Table**) together with their abbreviations.

**Fig 2:**
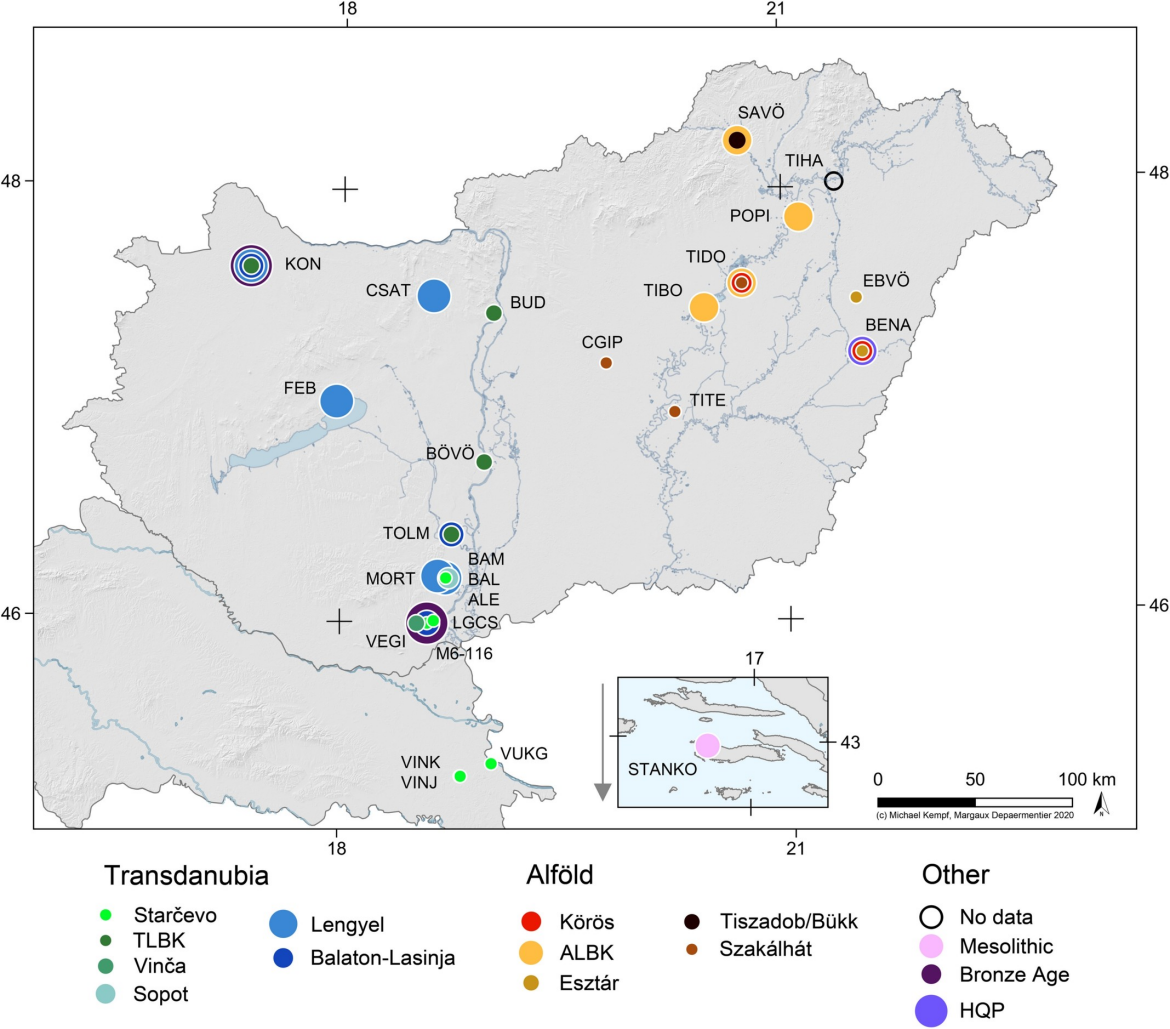
Sites where human dental enamel was sampled for oxygen isotope analyses. Some sites provided samples from several archaeological cultures that are represented by overlapping coloured dots. The full name of each site can be found in the Supplement (**Table A in S1 Table**) together with their abbreviations.

In this study, 718 human dental enamel samples from 55 Neolithic (and Chalcolithic) sites were collected for strontium and oxygen isotope analyses within the framework of the research project "*Bevölkerungsgeschichte des Karpatenbeckens in der Jungsteinzeit und ihr Einfluss auf die Besiedlung Mitteleuropas*" led by Kurt W. Alt and Eszter Bánffy. The further processing was realised by the cooperation 2014–2018 "*Das südungarische Sárköz-Gebiet im Neolithikum*", between the Institute of Archaeology, Research Centre for the Humanities in Budapest and the Roman-Germanic Commission of the German Archaeological Institute (DAI) (project leader E. Bánffy). The total sample includes Neolithic sites from the Carpathian Basin, from both Transdanubia and the Alföld, and a few comparative individuals from northern Croatia (**Figs 1 and 2, Table A in S1 Table**). The sampling process was carried out by Jakucs János and Anna Szécsényi-Nagy during 2008–2013 and permitted by the museum or scientific institution whose collection included the sampled anthropological material (**S2 Table**). The use of the samples was authorized by the aDNA Laboratory of the Institute of Archeology of the Hungarian Academy of Sciences to which the samples belong.

For the strontium isotope analyses, our study sample comprised 412 individuals from almost all selected Neolithic sites (**Fig 1 and Table A in S1 Table**), while 190 individuals from 28 sites (**Fig 2**) were analysed for oxygen stable isotopes (**Table A in S1 Table**). The discrepancies between the samples for strontium and oxygen isotope analyses are related to technical issues. Whenever possible, equal numbers of males and females were sampled from each burial community. About one-third of the individuals in each sample are children who could not be sexed. The morphological sex was determined by the method introduced by Éry and colleagues [30]. The anthropologists recorded metric and morphological characteristics, which are showing the sexual dimorphisms. These included eight skull, four jaw, and eleven postcranial characteristics [31]. The Transdanubian sample comprises individuals from the Starčevo, TLBK, Vinča, Sopot, Lengyel, and Balaton-Lasinja cultures, while the Alföld sample contains individuals from the Körös, ALBK, Esztár, Tiszadob/Bükk, Szakálhát, Tisza and Tiszapolgár groups; however, the two latter groups are not represented in the sample for oxygen stable isotope analyses (**Figs 1 and 2**). The total sample also includes Mesolithic (n = 2), Baden/Vučedol (n = 5), Avar (n = 1), Hungarian Conquest period (HQP) (n = 1) and undated (n = 14) individuals. Owing to their small sample size, non-Neolithic samples did not yield representative data and are therefore not discussed in this paper.

**Fig 3:**
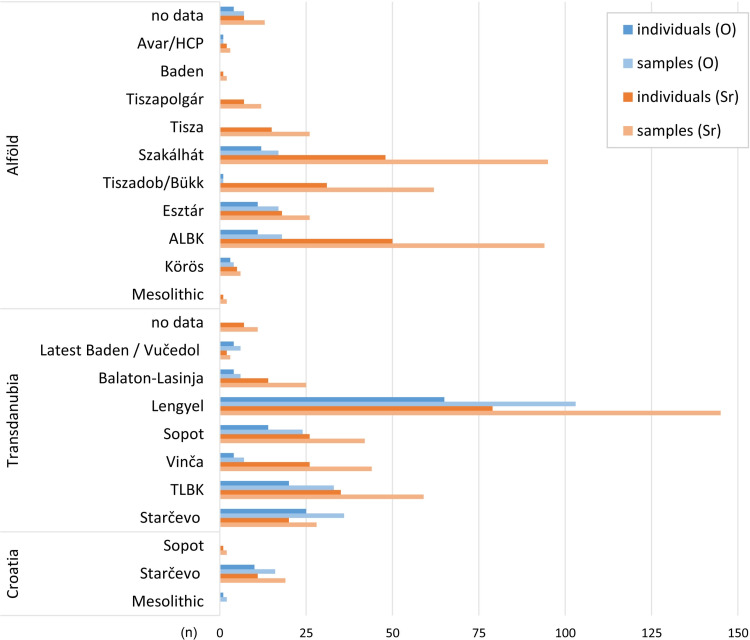
Number of individuals and corresponding dental enamel samples selected within the framework of this project for strontium and oxygen isotope analyses. They are organised by regions and archaeological cultures.

Given that some periods were clearly underrepresented in the sample (**Fig 3**), 183 published data from 14 additional Hungarian Neolithic sites [31–33] were added to complete the sample (**Table A in S1 Table**). By covering the entire Neolithic period in present-day Hungary, the datasets for strontium and oxygen isotope analyses enable the identification of parallel and diachronic trends in human mobility in Transdanubia as well as in the Alföld.

Whenever possible, at least two teeth were sampled per individual, usually corresponding to an early mineralising (deciduous tooth, M1/I/C or M2/PM) and a late-forming tooth (M2/PM or M3), which would provide insights into the successive stages of an individual’s childhood and related mobility patterns. More details about the sampled material and about baseline data are listed in **Tables A–C in S1 Table** [see also 12].

**Fig 4:**
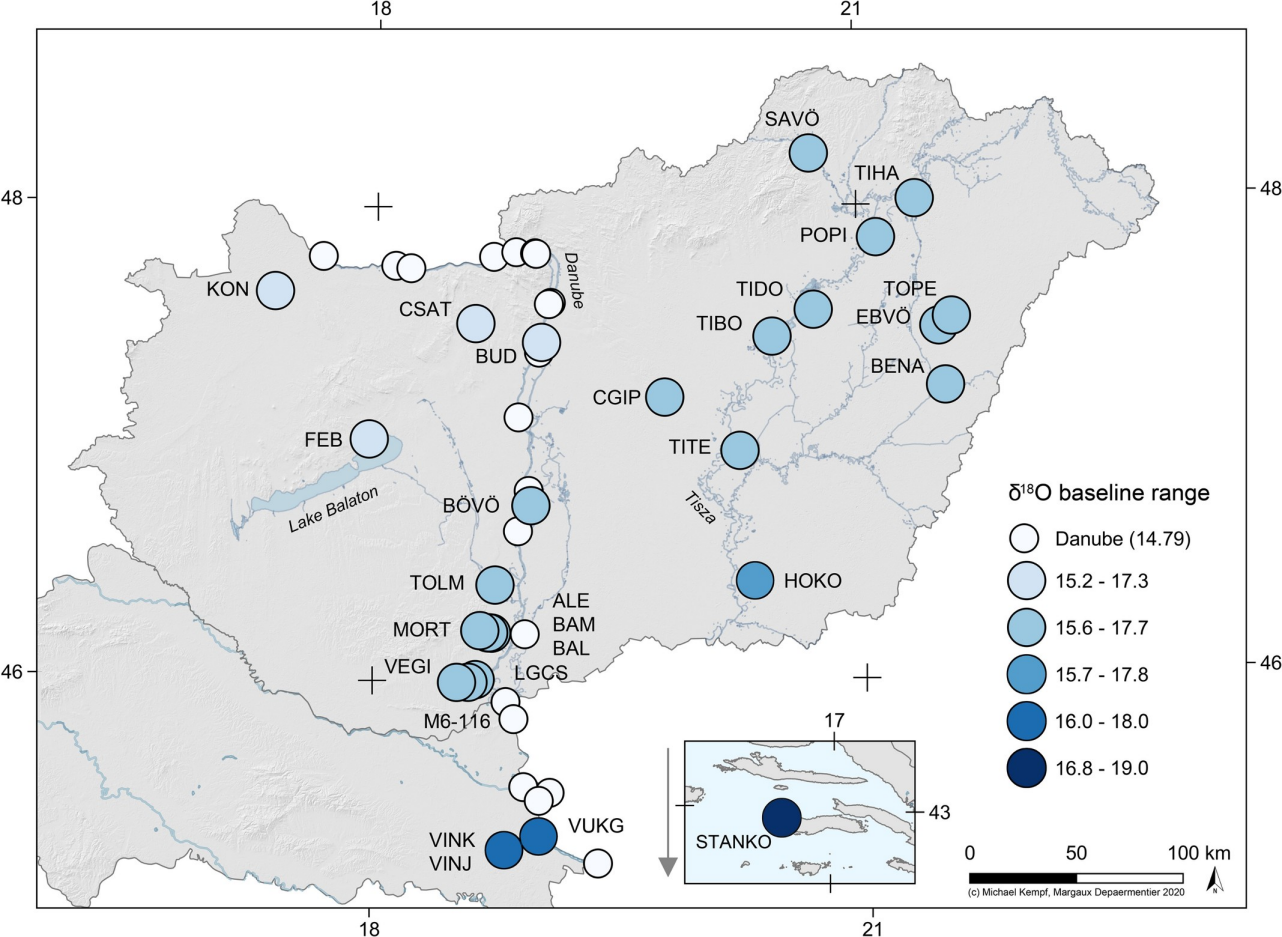
δ^18^O local baseline ranges at the different sites in the sample. The ranges were artificially fixed to ± 1 ‰ around the average δ^18^O value of modern precipitation data at each site (data source: OIPC (waterisotope.org, last accessed: February 2, 2020) [37–40]). Modern δ^18^O data of the River Danube in Hungary [64–66] were included on the map.

Furthermore, in order to determine a strontium isotope baseline for each site, samples were taken from 159 archaeological human bones from 42 sites, 103 archaeological faunal tooth enamels from 30 sites and 29 modern shells (including two *gastropoda* and 27 *bivalvia*) from 15 sites (**Tables B and E in S1 Table**). The faunal sample comprised three *Bos taurus*, three *Canis familiaris*, 29 *Ovis/Capra*, two *Cervus elaphus*, 54 *Sus domesticus* and eight *Sus scrofa* teeth. The determination of site-specific and micro-regional baselines using these data is presented and discussed in a separate paper [12]. Additional published data were used to complete this baseline dataset (**Table E in S1 Table**). In contrast, no baseline sample was available for the oxygen isotope analyses. Therefore, published data [34–36] and modern precipitation and hydrological data accessible on the WISER portal of the International Atomic Energy Agency (IAEA) homepage (see GNIP at https://www.iaea.org/services/networks/gnip and GNIR at https://www.iaea.org/services/networks/gnir), or generated by the Online Isotopes in Precipitation Calculator (OIPC) [37–40] accessible at http://wateriso.utah.edu/waterisotopes/pages/data_access/oipc.html were used to determine local oxygen isotope baselines (**Fig 4 and Table F in S1 Table**).

## Methods

### Strontium isotope analyses

The stable isotope ^87^Sr is formed by the radioactive decay of rubidium (^87^Rb, t1/2 ~ 4.7 x 1010 years) in the bedrock [41–44]. This local strontium becomes bioavailable during weathering processes, meaning that it enters the food chain through soil, water, and the vegetation [44–46] without significant fractionation between successive trophic levels [42, 45, 46]. Consequently, the ^87^Sr/^86^Sr values of human teeth are considered representative of the origin of the food consumed by one individual during the corresponding moment of its childhood [47]. A difference between the ^87^Sr/^86^Sr ratio of the dental enamel and burial place is interpreted as evidence of a change of residence or at least a change in the source of food during an individual’s life. Such conclusions require a reliable determination of the biologically available strontium in the local (or regional) habitat of the individuals [45, 48, 49]. In this study, strontium isotope analysis followed methods previously described by C. Knipper and colleagues [50, 51].

### Oxygen isotope analyses

In combination with strontium isotope analyses, parts of the samples were also analysed for stable oxygen isotope composition. In environmental water such as rain, snow, glaciers, rivers, lakes and groundwater, this ratio varies depending on environmental settings, geographical location, and the related climatic conditions [52–55]. Oxygen with a characteristic isotope composition enters the food chain–and hence the human and animal body tissues–partly through the vegetation, but mostly through the intake of environmental water [55, 56]. Therefore, the δ^18^O value in teeth and bones is directly related to environmental water and hence works as an indicator of human and animal geographic origin [54, 55, 57]. In this study, oxygen stable isotope analyses followed methods previously described by C. Knipper and colleagues [58].

Three standards were used with the TC/EA method (continuous flow mass spectrometry) for oxygen isotope analyses: the HAP, the NBS 120 c and the SUS-DAN (called SUD in **Table D in S1 Table**). As shown by C. Chenery and colleagues [59], the expected δ^18^O value for NBS 120 c (Ag_3_PO_4_) is approximately 22.3 ± 0.2 ‰ when using this method. The expected δ^18^O values for HAP and SUS-DAN are 17.1 ± 0.24 ‰ and 14.3 ± 0.23 ‰, respectively [58, 60]. The data were standardised to IVA (Ag_3_PO_4_). Both standards and human enamel were usually analysed three times–sometimes only twice–and the mean value was used for interpretations (**Table D in S1 Table**). The δ^18^O value for NBS 120 c was 22.2 ± 0.3 ‰ (n = 20, excluding the three outliers), the δ^18^O value for HAP was 17.12 ± 0.2 ‰ (n = 24) and the δ^18^O value for SUD was 14.19 ± 0.2 ‰ (n = 22). These results correspond to the expected values of these standards using the TC/EA methods (**Table D in S1 Table**).

### Baseline determination and implications

**Fig 5:**
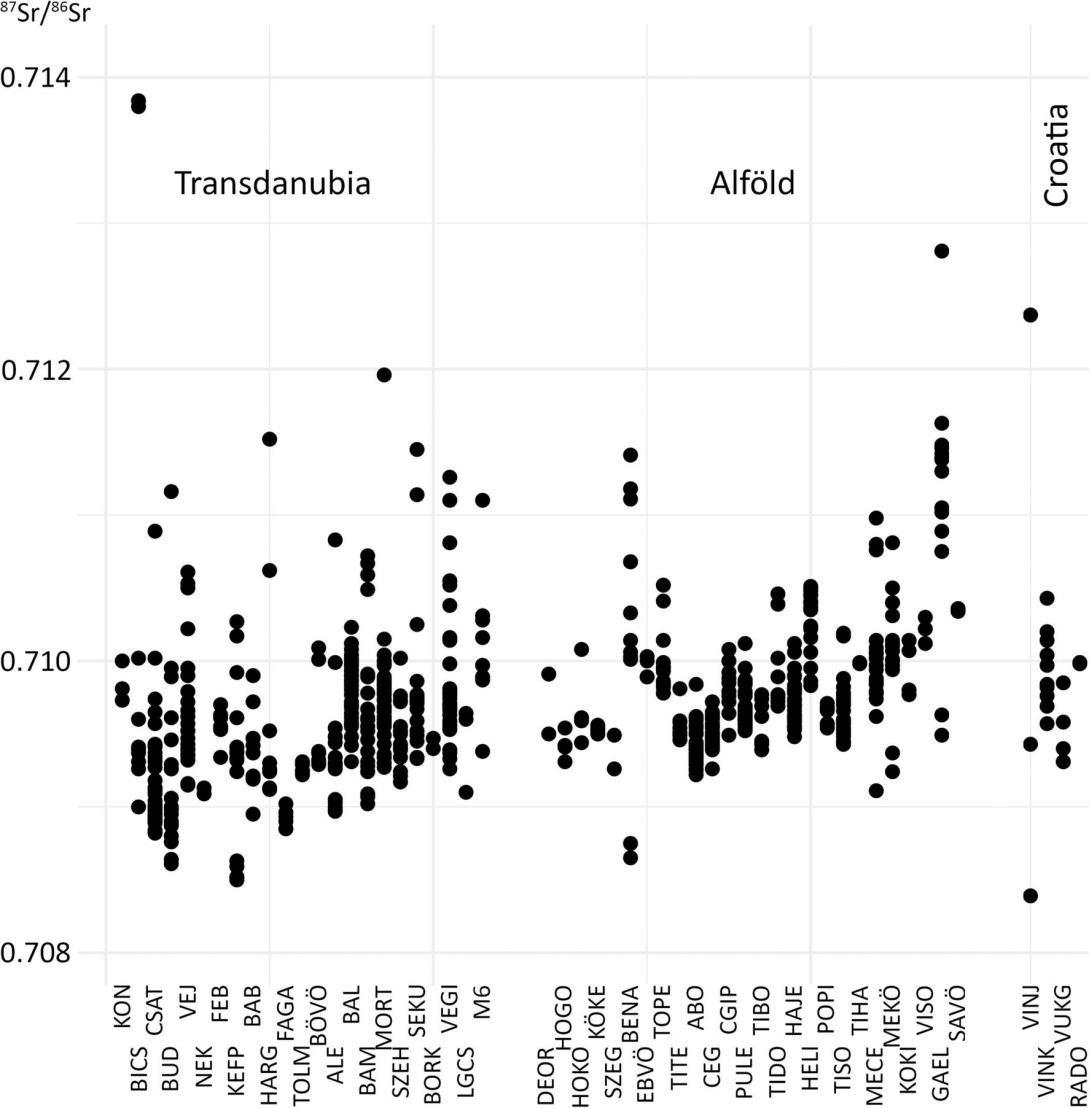
Human dental enamel ^87^Sr/^86^Sr ratios per site. The sites follow a geographical order: Transdanubia (north to south); Alföld (south to north). Croatian samples are listed separately.

Local or regional isotope baseline determination is the first requirement for interpreting human dental enamel isotope composition. In order to compensate for occasional small sample sizes, a new method integrating multivariate environmental data was applied, which is presented in a separate paper [12]. As demonstrated by the authors, this method enabled the proposal of both a site-specific and a wider micro-regional strontium isotope baseline for most of the studied sites (see **Tables B and E in S1 Table**). Consequently, this paper does not use the common “local” and “non-local” terminology for the interpretation of strontium and isotope data, but refers to the new classification introduced by Kempf and colleagues [12], which enabled the identification of site-specific, micro-regional and non-local individuals. By comparing the isotope composition of human enamel with the expected site-specific and/or micro-regional strontium isotope baseline range (see **Tables A–E in S1 Table**), it was possible to distinguish site outliers that may have originated from the broader area of the site (“micro-regional”) and site outliers that came from distant and environmentally distinct locations (“non-local”). In addition, sampling at least two teeth per individual also allowed considering the difference between early and late mineralising teeth for almost every individual. Individuals with an isotope composition matching the site-specific baseline range in the early-forming tooth but having a later mineralising tooth with a strontium isotope ratio falling outside the range were considered “mobile”. Furthermore, the normal variation in strontium isotope composition within a site could be calculated by adding two standard deviations to the average value of the difference between both deciduous teeth, between the deciduous tooth and the first molar, and between both first molars [58]. Therefore, when the difference between the ^87^Sr/^86^Sr ratios or δ^18^O value of one individual’s teeth exceeded the offset expected for the normal variation within a site, the individual could be considered “mobile” as well (see **Figs 5 and 7**).

Because of the lack of comparative baseline data, the oxygen stable isotope local range was calculated by adding plus or minus 1 ‰ to the long-term average δ^18^O value of modern precipitation at each site (**Figs 4 and S11 and S12**), which is the most usual span known from the literature for a local oxygen isotope baseline (e.g. [36, 58, 59, 61–63]).

In order to compare Neolithic human data with modern precipitation data and despite the fractionation of stable oxygen isotope during metabolic processes, the δ^18^O_Phosphate_ were converted into δ^18^O_Water_ using the following linear regression equation: ([67], 'superset').

$$\delta^{18}{\text{O}}_{\text{P}}=(33.49+\delta^{18}{\text{O}}_{\text{W}})/1.55$$

Similarly as for the strontium isotope analyses, every human dental enamel sample exhibiting an oxygen isotope composition that corresponds to the local baseline range was considered local and the others as site outliers. However, early-forming teeth such as deciduous teeth or first permanent molars are susceptible to showing a breastfeeding signal if the individual consumed breast milk (which is enriched in ^18^O) during the first months or years of its life [68]. Because the breastfeeding effect is known to produce an offset of up to 1.3 ‰, early mineralised teeth showing a similar offset compared to late mineralised teeth of the same individual were assumed to reflect this effect instead of mobility patterns [58, 68]. Individuals that exhibit larger or inverted offsets between the first permanent molar or deciduous teeth and the later mineralised tooth were considered “mobile”. In this study, oxygen isotope values could therefore represent three different mobility patterns: “local”, “mobile” or “non-local”. Because the composition of bioavailable oxygen isotopes is homogeneous across a broad geographical region (**Fig 4**), it is not adequate to use the term “site-specific” or the category “micro-regional” for the interpretation of oxygen isotope data. Finally, some individuals were only analysed for strontium isotope composition and a few others only for oxygen isotope composition. In these cases, the results were interpreted with a single data source: strontium or oxygen isotope analyses. When an individual provided samples on which both strontium and oxygen isotope analyses could be performed, both results were compared and added to each other in order to interpret the data (**S3 Table**). If the strontium and oxygen isotope data delivered contradictory results, the analysis that provided a non-local signal was used for the interpretation.

## Results

### Strontium isotopes

The ^87^Sr/^86^Sr ratios of human dental enamel range from 0.70839 to 0.71384, with an average value around 0.709714 for the whole sample, including the Croatian individuals (**Figs 5 and S5–S10**). The lowest value was found in a canine tooth of a medieval male individual (VINJ4) from the Vinkovci Jugobanka site in Croatia, while the highest value was found in the first permanent molar of an infant (BICS5) from the Sopot culture at Bicske-Galagonyás. The ranges and median values for males, females, subadults and indeterminate individuals by site are listed in the form of boxplots in the Supplement to this article (**S1 Fig**). Depending on the site and the archaeological culture, strontium isotope values varied widely between female, male and subadult individuals. However, because the sample size per site and, even more importantly, the sample size per archaeological culture at each site is usually very small, this study mainly focuses on the comparison of the different cultural groups represented by the whole sample (**S2 Fig**).

**S1 and S2 Figs** show that when considering only the sites that provided enough samples to consider differences between the sexes, females showed more variable strontium isotope data than males during the Starčevo, TLBK, Vinča, and Lengyel periods in Transdanubia and during the ALBK, Szakálhát and Tiszapolgár periods in the Alföld. In the Sopot group, males have more variable strontium isotope than females. In the Tiszadob/Bükk and Tisza groups, males and females show alternatively more or less variable strontium isotope data depending on the site. Moreover, the strontium isotope ratios of infants and adolescents do not necessarily correspond to narrower ranges as compared to adult individuals.

**Fig 6:**
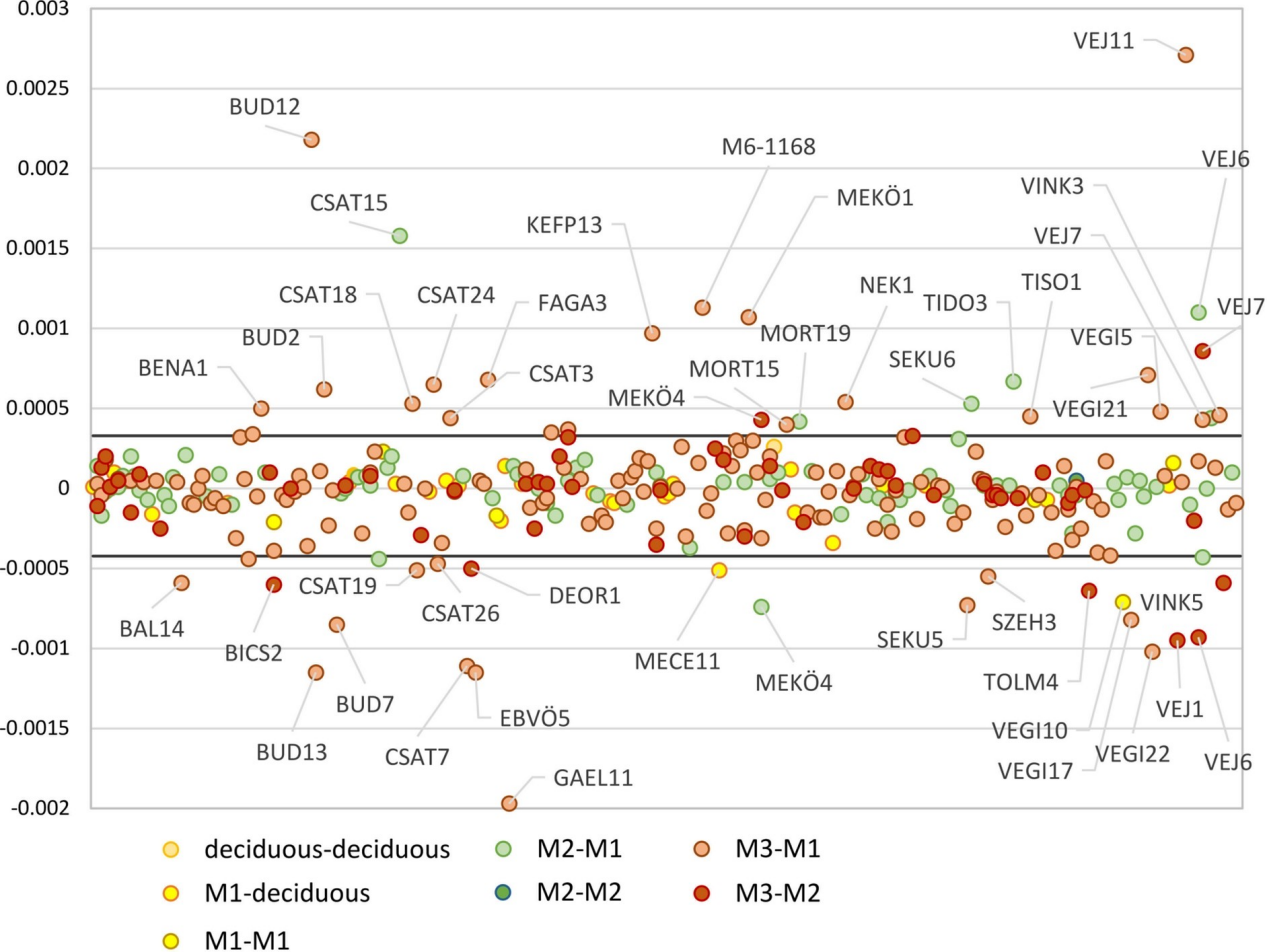
Differences in the ^87^Sr/^86^Sr between teeth from the same individuals. The normal variation in strontium isotope composition within the sites could be calculated by adding the two standard deviations to the average value of the difference between both deciduous teeth, between the deciduous tooth and the first molar, and between both first molars (range marked by the black lines). The labelled individuals show an offset that exceeded the normal variation within the sites, which could be related to mobility or to a significant shift in food sources during tooth formation.

In order to interpret the strontium isotope data, the human dental enamel ^87^Sr/^86^Sr ratios of each sample were compared to the site-specific and to the micro-regional baseline–if available–of the site [12]. The ratios were represented per cultural group and per site or site complex in several diagrams (**S5–S10 Figs**). Applying the considerations described above, the individuals could be interpreted either as site-specific, mobile, micro-regional, or non-local (**S3 Table**). An additional method for identifying mobile individuals involved the comparison of the offset between the ^87^Sr/^86^Sr ratios of different teeth from the same individual to the expected normal variation within a local area (**Fig 6**). When one individual showed an offset that exceeded this range, he/she was considered mobile (see labelled individuals in **Fig 6**).

### Oxygen isotopes

**Fig 7:**
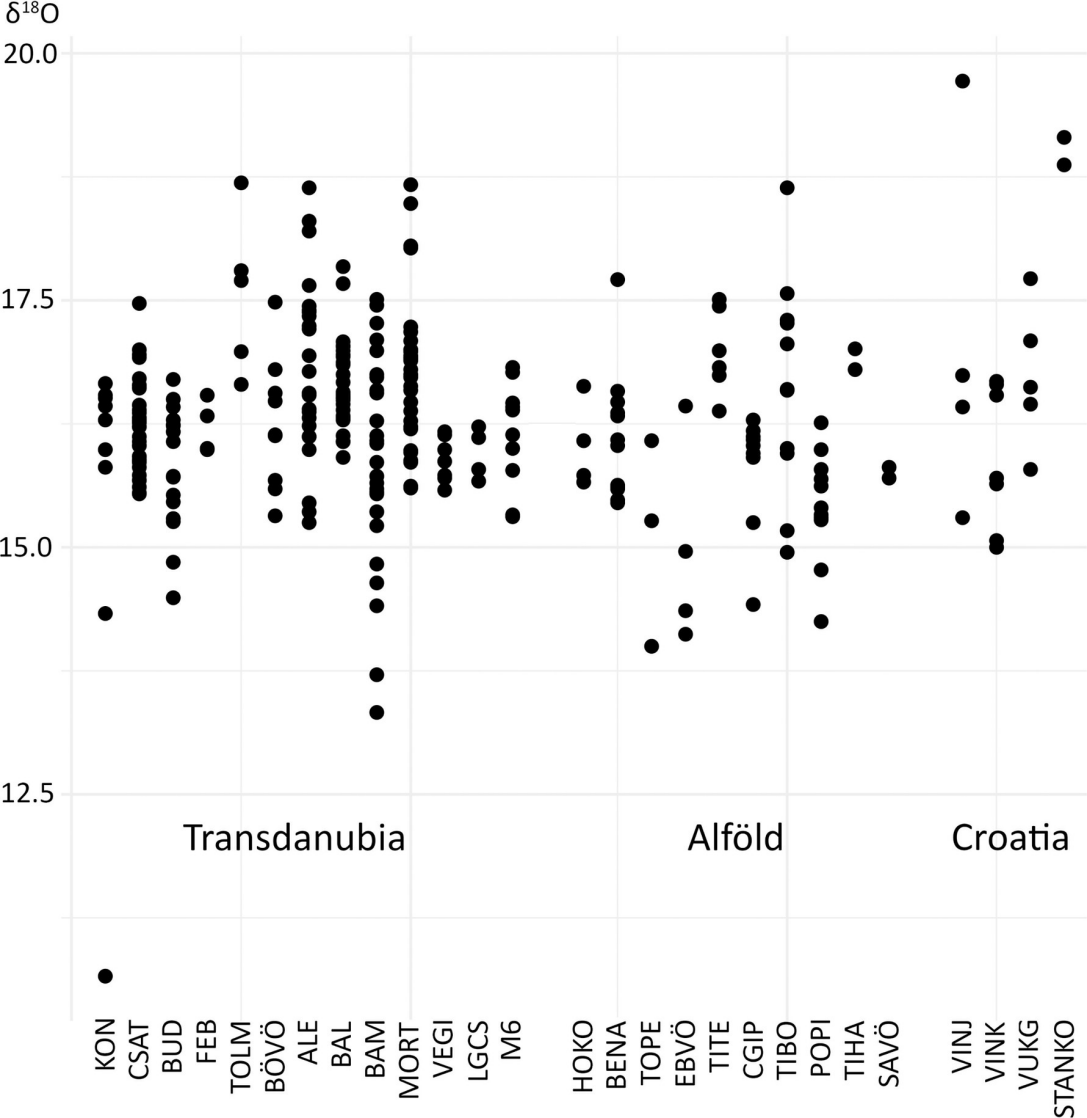
Oxygen isotope data (δ^18^O in ‰) of human dental enamel from Transdanubia, the Alföld and Croatia. The sites follow a geographical order: Transdanubia (north to south); Alföld (south to north). Croatian samples are listed separately.

The δ^18^O ratios in the phosphate of human tooth enamel (δ^18^O_P_) ranges from 10.66 to 19.72 ‰, with an average value of 16.28 ± 0.9 ‰ for the whole sample including the Croatian samples (**Figs 7 and S3, S4, S11 and S12**). The lowest value was found in the third molar of a Balaton-Lasinja female (KON2) from the Kóny 85 Enese site. The highest value was found in a canine tooth of a medieval male (VINJ4) from the Vinkovci Jugobanka site in Croatia. The ranges and median values for males, females, subadults and indeterminate individuals per site and archaeological culture are represented in the form of boxplots in **S3 Fig**. When including or excluding the early mineralising teeth that could be affected by breastfeeding effects, females, males, indeterminate adults, and subadults show widely varied oxygen isotope values depending on the site and the archaeological culture. By comparing the different cultural groups represented by the whole sample (**S4 Fig**), disparities were highlighted at the site rather than at the cultural level. For example, males and females showed distinct ranges at Bátaszék-Mérnöki telep (BAM), Alsónyék elkerülő (ALE), Mórágy-Tűzkődomb (MORT), Versend-Gilencsa (VEGI) and Budakeszi Site 8 Szőlőskert-Tangazdaság (BUD) in Trandsdanubia, as well as at Hódmezővásárhely-Kotacpart (HOKO) and Polgár-Piócási-dűlő (POPI) in the Alföld. In contrast, the males and females from the Bátaszék-Lajvér (BAL) and the Csabdi-Télizöldes (CSAT) sites showed generally comparable oxygen isotope values.

**Fig 8:**
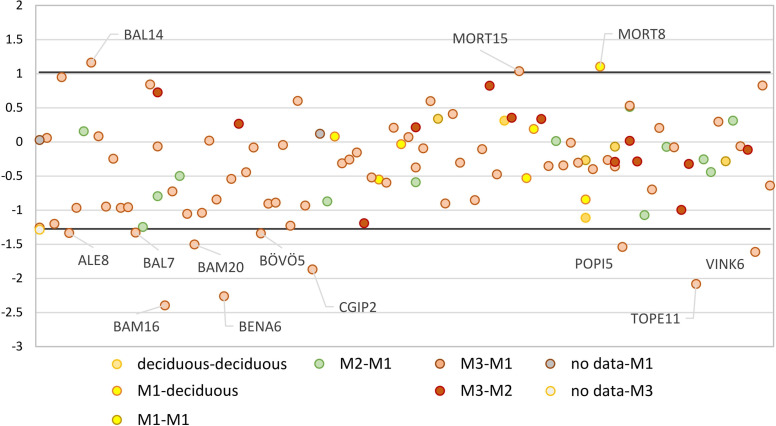
Differences in the δ^18^O between teeth from the same individuals. The normal variation in oxygen stable isotope composition within the sites could be calculated by adding two standard deviations to the average δ^18^O value of the difference between both deciduous teeth, between the deciduous tooth and the first molar, and between both first molars (range marked by the black lines). The labelled individuals show an offset that exceeded both the normal variation within the sites and the expected offset related to a potential breastfeeding-effect, which could therefore be related to mobility or to a significant shift in food sources during tooth formation.

The first step when interpreting the oxygen isotope composition of the samples was to compare the human dental enamel δ^18^O value with the local baseline range. The data were represented by archaeological cultures and by site or site complex in several diagrams (**S11 and S12 Figs**). Applying the considerations described above, each individual could be interpreted as local, mobile, or non-local (**S3 Table**). Mobile individuals were also identified by comparing the offset between the δ^18^O data of an individual’s different teeth to the locally expected normal variation in δ^18^O data, which was calculated with two standard deviations over the average difference between the δ^18^O data of two deciduous teeth, of the deciduous tooth and the first molar, and of two first molars of the same individual (**Fig 8**).

## Discussion

### Temporal and geographical differences

**Fig 9:**
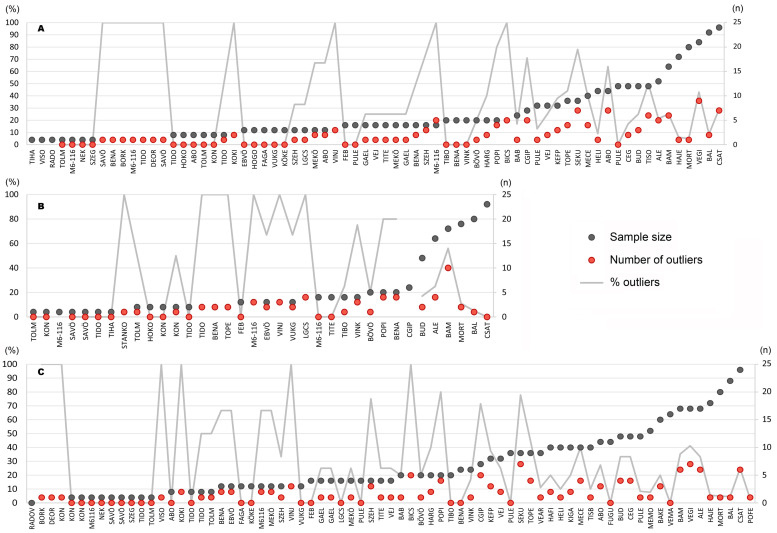
Comparison of the number (red dots) and the percentage (light grey lines) of site outliers with the sample size (dark grey dots) per site. Different archaeological cultures represented at the same site are illustrated separately in the diagram. A) represents the interpretation of strontium isotope data, B) represents the interpretation of oxygen stable isotope data, and C) represents the final interpretation combining strontium and oxygen stable isotope data.

Because the sample includes a large number of widely distributed individuals with inconsistent per-site data samples, this study focuses mainly on the consideration of the various cultural groups as determined in archaeological scholarship (see **Figs 1 and 2**). The interpretation of each individual’s data is available in **S3 Table** and their interpretation on the site level indicates a great variety of mobility disparities between sex and age categories at contemporaneous sites. However, no significant patterns related to the chronological or spatial criteria could be identified (**S21 Fig**), which can be explained by the mostly small sample size per site. By comparing the percentage of site outliers (corresponding to both non-local and micro-regional individuals) to the sample size at each site (**Fig 9**), it is obvious that extreme values (0 and 100%) mostly occur when the sample size does not exceed six individuals. Only six samples that include more than six individuals have no site outliers (**Fig 9A** PULE, n = 12, **Fig 9B** CSAT, n = 13, **Fig 9C** PULE, n = 9; FUGU, n = 11 and VEMA, n = 16). This highlights the problem of sample representativity.

**Fig 10:**
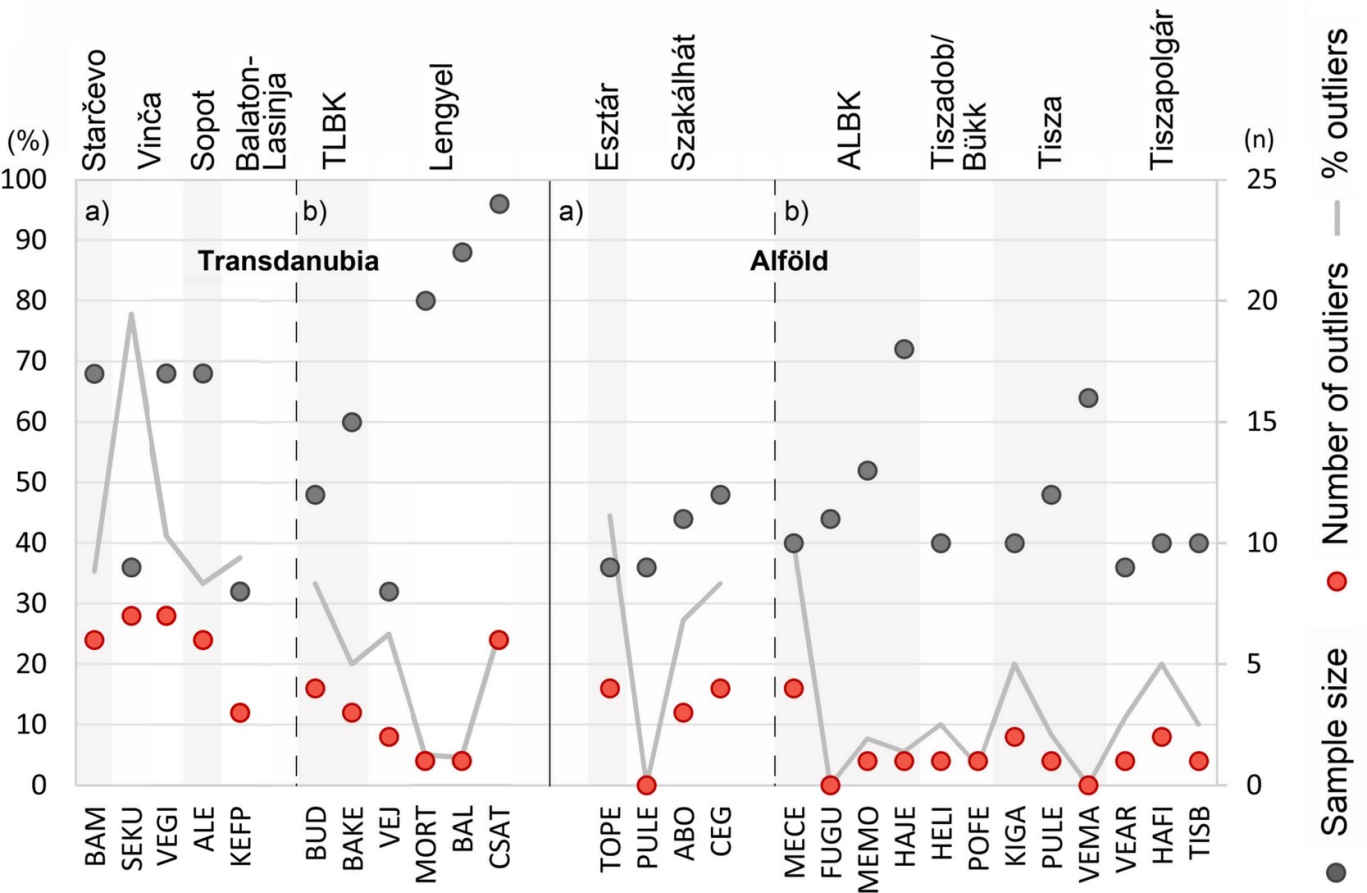
Comparison of the number (red dots) and the percentage (light grey lines) of site outliers with the sample size (dark grey dots) at the sites that provided least eight individuals. This diagram makes a distinction between archaeological cultures for which a large (a) and a small (b) proportion of site outliers is expected.

**Fig 11:**
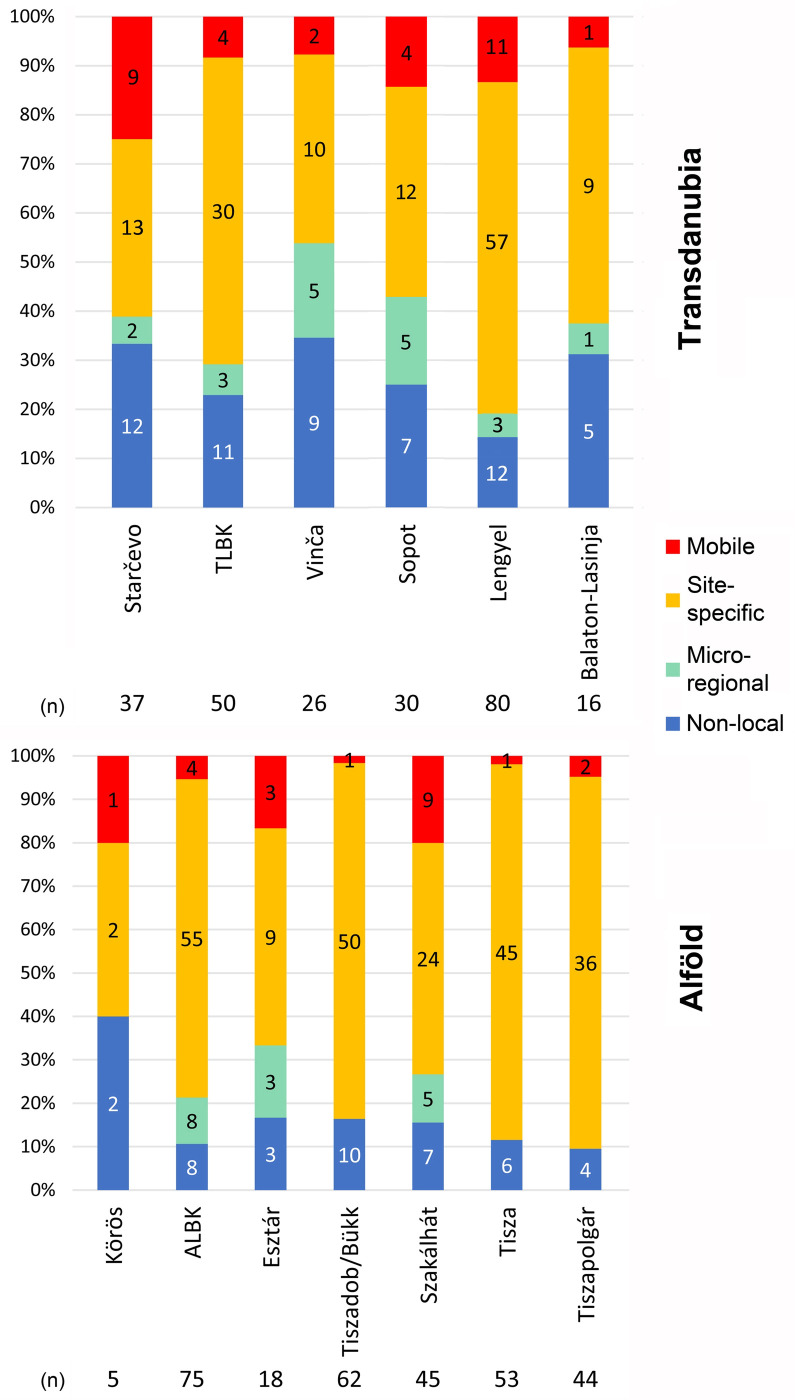
Number and percentage of site-specific, mobile, micro-regional and non-local individuals per archaeological cultures in Transdanubia and in the Alföld for both strontium and oxygen isotope analyses. The numbers (n) indicate the sample size for each cultural group over the whole sample. Because of similar cultural affiliations, the Croatian samples were included in the Transdanubian diagram.

Results based on a small sample size cannot be considered representative enough to be extrapolated on the site or the cultural group level. In archaeological research, however, large sample sizes are rarely available. In this study, only three sites yielded samples with at least 20 individuals. 21 sites provided samples with at least 10 individuals and six further samples include at least eight individuals. Only these samples are used for pattern recognition on the cultural group level (**Fig 10**). Furthermore, the integration of the data on the cultural group level enables handling larger sample sizes, which are more representative for each archaeological culture, except for the Körös sample, which includes no more than five individuals. The results allow for the hypothesis that the various Neolithic cultural groups show distinct mobility patterns (**Fig 11**).

At the onset of the Neolithic, the Starčevo cultural group in Transdanubia and the Körös group in the Alföld are characterised by a large proportion of site outliers (around 40%) (**Fig 11**), as would be expected from the Neolithisation narratives [1–4], although the small Körös sample size (n = 5) cannot be considered representative for the whole Körös cultural group. In this context, it is therefore not possible to confirm the assumption that the Körös group was mostly made up of immigrant individuals, although this is strongly indicated by the archaeological and aDNA evidence [1–4, 7, 10]. In contrast, this phenomenon is suggested by the more representative sample (n = 37) for Starčevo on both the site and the cultural group scale (**Figs 10 and 11**). During the ensuing LBK period, Neolithic communities had a decreased degree of residential mobility [2, 5, 69]. This assertion is confirmed by this study on the site scale in the Alföld, except for the ALBK site at Mezőkeresztes-Cethalom (MECE) (**Fig 10B**), while no clear tendency can be discerned in Transdanubia regarding TLBK sites, where the number and proportion of site outliers is nevertheless generally low. On the cultural group level (**Fig 11**), the TLBK and ALBK sample show small proportions of site outliers (under 30%) and can therefore be considered communities with mainly local activities. Mobility setting out from the LBK sites is another question and it is not measurable among the people who remained and spent their lives on their respective settlements. Furthermore, it is possible that mobility remains unnoticed in isotope composition of human dental enamel if it took place between areas with similar strontium and oxygen isotope values [12, 46, 70]. The proportion of non-local individuals might therefore remain underestimated. This applies for the whole study, because the Carpathian Basin is broadly covered by Pleistocene loess, loam, and Quaternary fluvial sediments and may hence be considered geologically and isotopically homogeneous over wide areas [31–33, 71].

In the late TLBK, new influxes from northern Croatia through the Sopot group and from Voivodina through the Vinča culture are archaeologically attested [16, 22]. The interpretation of the strontium and oxygen stable isotope data indeed shows a particularly high proportion of site outliers in the Vinča samples: between 40 and 80% on the site scale (**Fig 10A**) and over 50% on the cultural group level (**Fig 11**). This tendency is also obvious concerning the Sopot culture, with more than 40% site outliers on the cultural group level (**Fig 11**). However, only one Sopot site provided a relatively large sample size (see ALE in **Fig 10B** and [40]). In the Alföld, two different patterns can be observed concerning the Tiszadob/Bükk and Esztár groups of the developed ALBK: on both the site scale (**Fig 10**) and the archaeological culture level (**Fig 11**), the Tiszadob/Bükk sample reveals particularly low proportions of site outliers (under 10% at HELI and POFE), while the Esztár sample shows increased proportions of site outliers. This may be an indication of the different ways in which these communities merged with ALBK [17, 18, 69, 72], which will be discussed at greater length below. An increased proportion of site outliers can also be observed at two of the three Szakálhát sites (**Fig 10A**) and for the whole Szakálhát sample (**Fig 11**). This phenomenon is consistent with the archaeological record that shows that the Szakálhát groups are characterised by new features and extensive contacts [19, 20].

At the end of the Neolithic, Transdanubia is first marked by a decrease in mobility and external inputs during the Lengyel period (under 30%, see **Fig 11**), as can be seen especially at the BAL and MORT sites (under 10%) [see also 26] and, to a lesser extent, at the CSAT site (under 30%) in **Fig 10**. This can perhaps be taken to indicate that the South-East European impacts observed in the material record of the Lengyel culture can probably be ascribed to trade rather than to the arrival of new population groups. In contrast, the increased proportion of site outliers during the Balaton-Lasinja period (nearly 40%) (**Figs 10 and 11**) correlates well with the archaeological record, according to which a new Balkanic influx can be attested [1–4]. In the Alföld, the data show, as would be expected from the archaeological [69, 72], aDNA [6–8] and further strontium isotope data [31–33], that the Tisza and Tiszapolgár groups are characterised by a particularly small proportion of site outliers (usually under 20%). These communities had thus spatially restricted daily activity areas. The assumed Balkan influx during the Tisza culture is not supported by this dataset.

The results of the strontium and oxygen stable isotope analyses and the interpretation of the data are generally consistent with the results of aDNA analyses conducted by Anna Szécsényi-Nagy and Victoria Keerl at the same sites, also within the framework of this project [6–8]. This project thus enables to retrace the fluctuations in mobility rates during the Neolithic in both Transdanubia and the Alföld. Another tendency illustrates that Transdanubian cultural groups usually have larger proportions of site outliers than the groups in the Alföld, even regarding the LBK groups (**Figs 10 and 11**). This can in all likelihood be explained by the geographical location of Transdanubia on the direct continental route of European Neolithisation that linked South and North in this part of Europe [7] and to the repeated Balkan inputs in Transdanubia [16, 22, 73–75], or to more extended social and economic networks [see 26]. Moreover, Transdanubia is geologically more diverse, so non-locals or mobiles individuals are more likely to be detected [31–33, 71]. The trend is therefore not necessarily cultural but could be triggered by the distinct environmental settings.

### Different types of mobility

Three different types of mobility can be distinguished through the application of the abovementioned methods. First, it is possible to identify individuals who were born at the site but were particularly mobile during their life. These individuals either have a site-specific signal in their early mineralising and a site outlier signal in their late-forming tooth, or have a large offset regarding the isotope composition of their different teeth. This means that some locally born individuals were mobile during their lives and returned to the home site at some later time. Usually, only two teeth were sampled in this study, which enables the determination that an individual left after the mineralisation of the earliest sampled tooth (usually the M1, with site-specific signal) and before the formation of the latest sampled tooth (usually the M3, with site-outlier signal). At Veszprém-Jutasi-Munkácsy út (VEJ), however, two individuals were sampled for the first, the second and the third molar. The second permanent molar of an adult female (VEJ6) shows a site outlier ^87^Sr/^86^Sr ratio–which could correspond to a micro-regional value–and the first and third molars show a site-specific ratio (**S6A Fig**). This could mean either that she changed her place of residence or her dietary habits when she was five or seven years old. Moreover, another adult female (VEJ7) has a site-specific ^87^Sr/^86^Sr ratio in both the first and the second permanent molars, while the third molar exhibits a site-outlier–but probably micro-regional–signal. She could have left her place of birth or changed her dietary habits when she was about seven years old; however, information about her later place of residence or the time of her return remain unclear. Although this region is very heterogeneous and the results could be interpreted in different ways, these observations clearly indicate that sampling several teeth per individual offers a more accurate insight into mobility patterns. Moreover, they are consistent with demographic and social mobility patterns assumed for prehistoric communities, in which children assume essential social and economic responsibilities at an early age (e. g. [58, 76]).

The second type of mobility corresponds to site outliers that probably originated from the immediate or (micro-)regional surroundings of the site. Given the determination of a micro-regional strontium isotope baseline [12], it is not only possible to distinguish individuals with site-specific from individuals with site-outlier isotope values, but also to divide site outliers into two categories: individuals with an isotope composition matching the micro-regional, and individuals that have neither site-specific nor micro-regional signals. Different or distant geographical areas can often have similar strontium and/or oxygen isotope compositions [12, 46, 70, 77], meaning that individuals with micro-regional signals could have originated from a distant region. However, the interpretation in this study is based on the principle that the nearest region with a corresponding isotope composition is considered the most likely place of origin (e.g. [63, 78]. The third type of mobility is represented by non-local individuals, who have neither a site-specific nor a micro-regional, but a non-local isotope composition in their teeth. They originated from a distant or geographically distinct region compared to the site and its surroundings and highlight spatially large-scale mobility patterns (e.g. by marriage or migration events).

The Starčevo and Körös archaeological groups show the overall highest number of mobile and site-outlier individuals (**Fig 11**). These communities were therefore demographically very dynamic. The high proportion of non-local individuals in the Körös and Starčevo cultural groups would be consistent with the archaeological “Neolithisation paradigm” positing that the first Hungarian Neolithic groups were predominantly “immigrants” [1–4] if the sample would correspond to the first generation of settlers. This would also fit in nicely with the results of aDNA analyses conducted on the same Neolithic sites, which highlighted the important role of immigrants in the composition of these communities [6]. However, it is not possible to identify the difference between initial settlers and later generations in the sample, so the interpretation of the non-local people in terms of large-scale migration and Neolithisation processes cannot be tracked with absolute certainty. According to the archaeological evidence, the first generation of Starčevo and Körös groups can be correlated with the expansion of the Anatolian-Balkanic agricultural civilisation in the 6^th^ millennium cal BC [1–4]. However, Borič and Price [79] and Voerkelius *et al*. [80] have demonstrated that the Balkans correspond to a wide area characterised by considerably differing ^87^Sr/^86^Sr ratios. Consequently, almost every non-local individual could theoretically be of Balkanic origin when considering only their human dental enamel ^87^Sr/^86^Sr data. In this context, oxygen isotopes work as a more suitable indicator (**Fig 4**), but the potential breastfeeding-effect that influences the oxygen isotope composition of deciduous teeth and first molars prevents the identification of the place of birth of non-local individuals [68]. Nevertheless, the oxygen data of non-local Starčevo and Körös individuals (BAM8, BAM10, BAM18, BAM20, M6-1164 and BENA3) do not match the expected Balkan values and would rather indicate a northern or alpine origin [37–40]. Consequently, this study confirms that a considerable part of the site outliers was not only non-local, but also non-regional, and illustrates the important role of supra-regional mobility in the Körös and Starčevo cultural groups. However, the origin of these non-local individuals and the temporality of this mobility cannot be determined with absolute certainty. Furthermore, social and economic parameters played an important role in this high mobility rate, as highlighted in the next part of the discussion.

As mentioned above, the Transdanubian and Alföld LBK communities are mostly site-specific and the mobility rate within the local area remains low (**Fig 11**). It is nevertheless noteworthy that the majority of TLBK and one-half of the ALBK site outliers do not originate from the micro-region, but from a more distant place. This shows that despite the possibly restricted daily activity area, some external inputs participated in the formation and development of LBK communities. In this case, it would be important to know whether this phenomenon concerns one specific LBK generation or whether it spans the entire LBK period. In Transdanubia, the Vinča group is again characterised by an increased mobility rate, where micro-regional and, above all, supra-regional inputs played a major role in the formation of the communities (**Fig 11**), which would be consistent with the archaeological models if it would correspond to the first generation of settlers, which is however not possible to determine. A Balkanic origin is also assumed for the Vinča site outliers [81], but this cannot be conclusively verified by the analysed strontium and oxygen isotope samples. The same holds true for the Sopot culture [16, 22]. However, an important part of Sopot site-outliers probably originated from the micro-regional surroundings of the sites–when assumed that the nearest region with comparable isotope composition is considered the most likely place of origin. Moreover, the Sopot communities were characterised by a relatively high proportion of mobile individuals within the local area. It is nevertheless possible that the yet already large amount of non-local individuals could be revised further upwards at the expense of the micro-regional and/or mobile categories. In the Alföld, the later ALBK groups–Esztár and Tiszadob/Bükk–have distinct mobility patterns. Esztár communities show a higher proportion of mobile and site-outliers individuals, indicating that the group is as much a blend of micro-regional as of supra-regional inputs, whereas the Tiszadob/Bükk group includes mainly site-specific individuals and an equal proportion of non-local individuals compared to Esztár, without any micro-regional inputs (**Fig 11**). The nevertheless comparatively high proportion of non-local individuals in the Tiszadob/Bükk sample could correspond either to ALBK movement towards the Bükk and Mátra Mountains [17, 82] at the end of the Neolithisation processes in the Upper Tisza region, which would be consistent with the archaeological [17], anthropological [83], and aDNA [6–8] record, or to Mesolithic inputs, which would also correlate with archaeological [84] and aDNA data [6, 7]. This is however impossible to identify the settler generations in the sample. According to the archaeological evidence, the Esztár culture had received impulses from the neighbouring Partium area of western Romania [74]. However, no origin could be determined either and aDNA analyses can answer this question for individual samples.

At the end of the Neolithic, decreased mobility rates, can be seen among the Transdanubian Lengyel communities, although the comparatively large proportion of non-local individuals highlights supra-regional inputs that could be related to the archaeologically assumed influx from the south [24, 25]–if corresponding non-local individuals belong to the first generation of settlers. In the Alföld, the Szakálhát groups are generally more mobile compared to Lengyel communities, but around one-half of the site outliers are classified as micro-regional (**Fig 11**). This highlights the important role of both small-scale mobility patterns and supra-regional inputs in the formation of these cultural groups, which is consistent with the current archaeological models [19]. Regarding the Tisza and Tiszapolgár groups, the low proportion of non-local individuals is not enlarged by regional inputs and the mobility rate remains generally low, confirming that the daily activities of Tisza and Tiszapolgár communities were mostly organised in a spatially restricted local area. In Transdanubia, however, the Balaton-Lasinja cultural group is characterised by a large number of site outliers, which mainly correspond to non-local individuals. This illustrates the important role of supra-regional inputs in the Balaton-Lasinja cultural groups, which match the archaeological assumptions. In contrast, micro-regional inputs played a minor role in the Balaton-Lasinja communities. However, the assumed Balkanic origin [73] cannot be verified.

### Residence patterns

**Fig 12:**
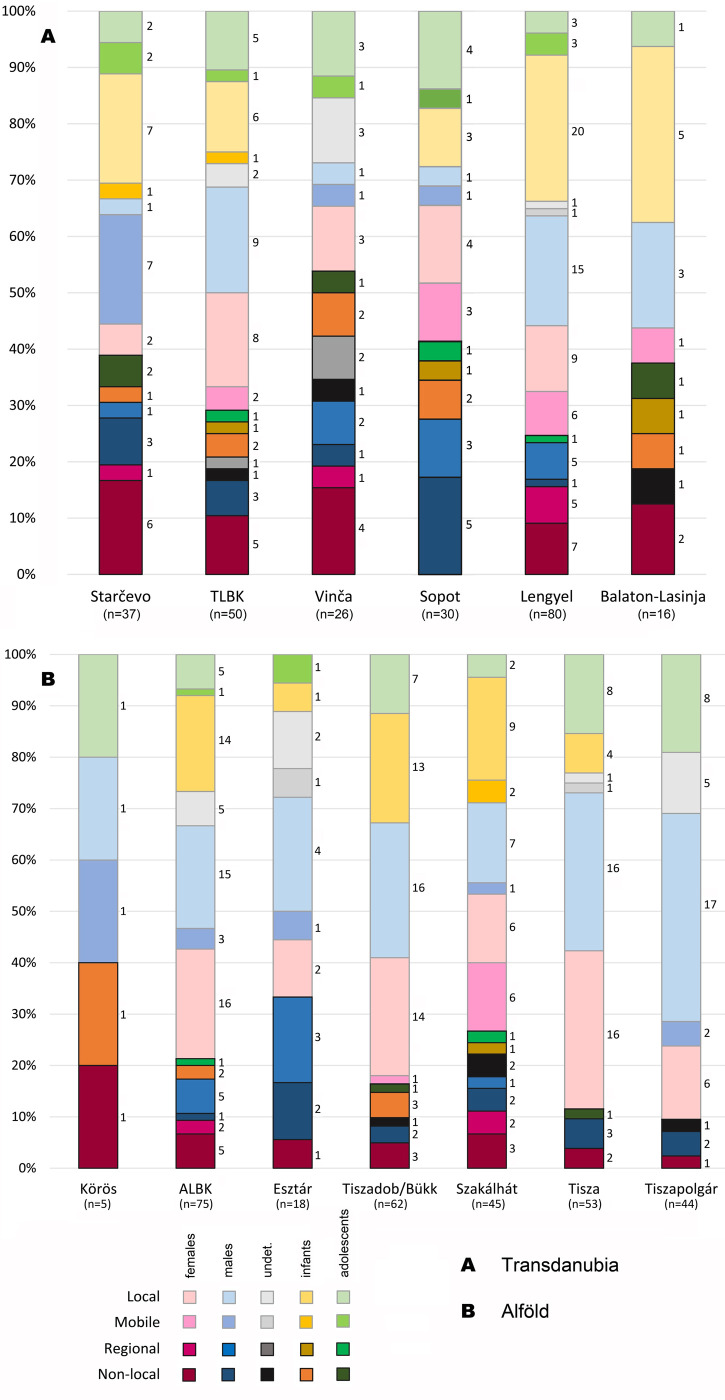
Percentage and number of non-local, micro-regional, mobile and site-specific males, females, indeterminate adults (undet.), infants and adolescents in Transdanubia (A) and in the Alföld (B). The data are presented by archaeological culture.

Differences can also be noted between sex and age categories at each site (see **S1–S20 Figs**) and regarding each archaeological culture (**Fig 12**). Depending on the cultural group, site-outlier individuals were alternatively mostly males or mostly females. Therefore, no regular partnership patterns can be observed in Neolithic Hungary. At the onset of the Neolithic, the Starčevo culture had higher proportions of non-local and micro-regional females compared to males, indicating that these groups were probably organised patrilocally. This assumption suggests that the large number of non-local Starčevo individuals could rather be related to extensive marriage networks than to Neolithisation processes [see also 26]. The widespread homogeneity of the material culture further suggests that these large-scale social networks were probably linked to considerable economic fluxes [2–3]. The Körös sample from the Alföld, however, is too small to enable any reliable conclusions regarding specific exogamy rules. The TLBK sample also highlights the probability that LBK communities were generally organised patrilocally, which is consistent with the results of the aDNA analyses [6–8], and with the results of previous strontium isotope analyses on LBK samples [85, 86]. However, the ALBK sample has approximately as many female as male site outliers and cannot confirm this model for the Alföld LBK communities. The same tendency can be noted regarding the Tiszadob/Bükk cultural group, while in the Esztár sample, site outliers are significantly more often males than females, suggesting that Esztár groups were matrilocal communities.

A similar pattern can be observed in Transdanubia, where the Sopot cultural group has solely male site outliers (**Fig 12**). This tendency, namely that Sopot males are more likely to move to other locations than females in a probable exogamy context was also attested in aDNA analyses [6–8] and would suggest that the high proportion of non-local individuals rather reflect large-scale marriage or economic networks than Neolithisation processes. In contrast, isotope analyses indicate that the Vinča cultural group was probably made up of patrilocal communities. Regarding the Transdanubian Lengyel group, the aDNA analyses indicated an increased paternal diversity [6–8]; however, the isotope data show a higher site outlier rate for females than for males (**Fig 12**). In the Szakálhát group in the Alföld, there are approximately as many female as male site outliers, with slightly more female site outliers, which does not permit any conclusions on residence patterns for the Szakálhát communities. Similarly, the number of Tisza and Tiszapolgár site outliers is too small to highlight specific exogamy rules. The Transdanubian Balaton-Lasinja group has a higher proportion of non-local and micro-regional females compared to males, suggesting that these were probably patrilocal communities. This assumption is consistent with the results of aDNA analyses performed on the same sample [6–8].

In the Alföld, non-local infants are only attested in the Körös, ALBK, and Tiszadob/Bükk cultural groups (in small proportions) and one regional infant is identified in the Szakálhát sample. In Transdanubia, each cultural group has at least one non-local infant, except for Lengyel. Otherwise, infants were mostly born on the site, which is hardly surprising, given that we can expect that children who died in the first months of their life did not have the time to change their residence before passing away. This proves the suitability of isotope data from young children’s dental enamel as an indicator of the local composition of bioavailable strontium isotopes (e.g. [12, 71]). In contrast, infants’ oxygen isotope data are probably not representative for the local signal because they are susceptible to be biased by the breastfeeding effect [68]. The presence of non-local children, however, indicates that at least some Neolithic families were (possibly seasonally) mobile, even though there were young children (infants) in the group. The infant sample size is nevertheless too small to enable the recognition of precise patterns depending on the sites or the cultural groups. Furthermore, most of the archaeological cultures show at least one non-local or micro-regional adolescent. Nevertheless, the site outlier rate remains low for adolescents and for Neolithic subadults in general. An increased mobility within the micro-regional area around the site can nevertheless be noted for adolescents compared to infants.

When juxtaposing the results of the strontium and oxygen isotope analyses with the archaeological record, no apparent patterns emerge relating to mobility and burial practices. Three examples can be cited to illustrate this assertion. The first concerns the Tiszadob/Bükk site at Tiszadob-Ókenéz (TISO), where two of the three sampled individuals that were buried in single graves [87] had non-local isotope values in their dental enamel (TISO3, TISO7), and a third was born locally (TISO15). Moreover, three individuals (TISO1, TISO4, TISO6) buried in a common grave pit exhibited non-local isotope compositions, while the other individuals in this pit had local isotope values. This would imply that an individual’s origin had no noticeable influence on the burial treatment in this Tiszadob/Bükk community. At the Abony site (ABO), the four Szakálhát individuals buried with Spondylus beads [88] were either mobile or non-local (ABO5, ABO13, ABO15, ABO17). However, the two other non-local Szakálhát individuals (ABO16, ABO19) had no grave goods and neither were they accorded a distinct burial treatment differing from the other members of this community. At the Lengyel site of Mórágy-Tüzkődomb (MORT), no specific pattern could be recognised when comparing the different grave good combinations [89] to dental enamel isotope composition. The burial of the single non-local female (MORT1) did not differ significantly from the rest of the group, while the single adolescent that was buried in an extended position (MORT8) was born locally and probably did not leave the site during its lifetime. The burial practices during the Neolithic in Hungary were therefore not significantly influenced by the origin or mobility habits of the various individuals [see also 26]. This assumption only concerns this study’s sample and should be tested for other European Neolithic communities.

### Combining strontium and oxygen stable isotope analyses

The first requirement for the interpretation of isotope data is the determination of a reliable baseline. Concerning strontium isotopes, Kempf and colleagues [12] showed that despite the lack of baseline data at many sites, the identification of comparable environmental settings enabled the combination of baseline samples from different sites and increasing the reliability of site-specific baselines. Furthermore, even sites located close to each other are likely to have fairly distinct environmental settings and therefore distinct site-specific baselines. The combination of distinct site-specific baselines within a site complex allowed suggesting a common micro-regional strontium isotope baseline for the area that includes those sites [12]. Similar issues concern oxygen stable isotopes. Since the baseline ranges were artificially fixed to ± 1 ‰ around the average δ^18^O value of modern precipitation data at each site, it is realistic to assume that the ranges could be lower or higher as suggested in this study. As shown by the OIPC European map that presents annual average δ^18^O data (accessible at http://wateriso.utah.edu/waterisotopes/pages/data_access/figures.html), the local isotope composition is generally homogeneous across present-day Hungary. This study confirmed that despite small regional disparities, the baseline ranges are consistent over large geographical areas (see **Fig 4**). This also means that it was not possible to distinguish between a site-specific and a micro-regional baseline. Consequently, individuals with a late-mineralising tooth with non-local δ^18^O data can also be regarded as “non-regional”.

Nevertheless, using these strontium and oxygen isotope baselines implies that modern data are comparable with Neolithic conditions. Even so, it remains difficult to determine the actual extent of the activity ranges of Neolithic groups around their sites [90], or to measure the actual effect of their cooking habits on the oxygen isotope composition of their tissues [91–93]. Furthermore, environmental settings consist of mixtures, transfers, smooth transitions, and overlaps [42], meaning that the limit values of strontium and oxygen isotope baseline ranges should be fuzzy (**S5–S12 Figs**). Consequently, if an individual exhibits an isotope composition close to the baseline range limits, the interpretation depends on the comparison with the isotope data of the rest of the group. At the Budakeszi Site 8 Szőlőskert-Tangazdaság (BUD) site for example (**S5B Fig**), the first permanent molar of a female (BUD2) and the third molar of an adolescent (BUD7) have strontium isotope ratios close to the lower baseline range limit. But because the differences compared to the rest of the site-specific group do not exceed 0.00015, they are not considered site outliers. However, the isotope composition of another adolescent’s (BUD9) first molar is right on the upper limit of the baseline range, but less consistent with the rest of the site-specific group and is therefore classified a site outlier.

The combination of strontium and oxygen isotope data, if available, provides more precise results than a single type of analysis would deliver. At the ALBK site of Polgár-Piócási-Dűlő (POPI), for example, only the first permanent molar of a female (POPI1) exhibits a site-specific ^87^Sr/^86^Sr ratio. The other four POPI individuals are site outliers that could nevertheless have originated from the micro-region around the site. This surprisingly high number of site outliers is confirmed by the oxygen isotope analyses. However, the δ^18^O data suggest that these non-local individuals probably did not originate from the direct surroundings of the site. One male (POPI5) in particular could have originated from a more distant place. Another example can be found at Ebes-Zsong-völgy (EBVÖ). The teeth of the three EBVÖ individuals show consistent site-specific strontium isotope compositions. Nevertheless, the only local δ^18^O value is found in a third permanent molar of the female (EBVÖ4), while the values of the two male individuals exhibit a non-local δ^18^O origin. Given that geographically distant areas can have similar strontium isotope values [12, 46, 70, 77], we deemed the male’s oxygen isotope data to be of greater value for classifying these as non-local. In general, however, this study found that the oxygen and strontium isotope data were in agreement for the majority of the sampled individuals.

## Conclusion

This study presents the strontium and oxygen stable isotope data of one of the largest Neolithic samples in Europe, whose assessment reveals the development of various mobility patterns over approximately 2000 years in both Transdanubia and the Great Hungarian Plain. Despite the sometimes small sample size for each site, it is nevertheless possible to interpret the data on different scales, from the individual and the burial community to the archaeological cultural group.

In Hungary, the Neolithic was very diversified in terms of the history of individual and group mobility. The migration and mobility models constructed within the framework of strontium and oxygen stable isotope analyses suggest that Neolithic communities were mostly organised on a local or at least a micro-regional scale, especially in the Great Hungarian Plain, which suggests a relatively homogeneous population structure. Small-scale mobility patterns involving both males and females to varying degrees within the local or micro-regional area around the site could be observed for nearly every Neolithic cultural group. For example, there are indications of patrilocality in the Starčevo, TLBK, Vinča, Lengyel and Balaton-Lasinja groups, while the Sopot and Esztár cultural groups can rather be considered matrilocal communities. Strontium and oxygen stable isotope analyses also highlighted large-scale mobility patterns, especially in Transdanubia. The particularly high number of non-local individuals in the Körös and Starčevo groups would only confirm the currently accepted Neolithisation process that was accompanied by extensive population movements if the sampled individuals corresponded to the first generation of settlers, which is not possible to attest. This influx was already over during the Hungarian LBK, while the Vinča and Balaton-Lasinja cultural groups in Transdanubia again showed higher proportions of non-local individuals, reflecting considerable new inputs from more distant regions. The archaeological record would suggest that they most likely moved along the Danube valley from the northern Balkanic area, although this could not be verified by this sample. In the Alföld, the Esztár culture also shows an increased proportion of supra-regional inputs. According to the archaeological evidence, the Esztár culture had received impulses from the neighbouring Partium area of western Romania.

However, it must also be borne in mind that large tracts of the Hungarian lowlands are covered with loess soils, meaning that their strontium isotope composition can be very similar despite the greater geographical distance between particular areas. Therefore, it can be assumed that non-local individuals are not recognised when the place of origin and destination have similar strontium isotope ratios. In this context, oxygen isotope values are a good complement to the strontium isotope values, although the data from early mineralised teeth cannot be used because of the breastfeeding effect. In sum, the results of the strontium and oxygen stable isotope analyses presented in this paper revealed further insights into the complex social and economic organisation of Neolithic groups that could be put into perspective with archaeological and aDNA data. The disparities between adult males and females regarding mobility patterns and the fact that it is not possible to identify settler generations in the sample suggest that high proportion of non-local individuals could be in some cases rather related to extended marriage, social, and economic networks than to Neolithisation processes, which also explains the widespread homogeneity in material culture of the corresponding Neolithic groups. Furthermore, no significant correlation between the strontium and oxygen isotope data and the grave goods or burial context is attested in this sample, suggesting that the variability of isotope ratios cannot be explained by social differences, and that local and non-local individuals did not receive differential mortuary treatment. Finally, the large total sample considered in this study not only provided outstanding results regarding small- and large-scale mobility patterns, but also delivered invaluable information on the organisation and dynamics of the various Neolithic communities discussed here.

## Supporting information

S1 Table Data tables A–F. Table A, Description of the samples; Table B, Details on the samples for strontium isotope analyses; Table C, Details on the samples for oxygen stable isotope analyses; Table D, Standards for oxygen stable isotope analyses; Table E, Comparative data for strontium isotope baselines in Hungary; Table F, Comparative data for oxygen isotope baselines at the studies sites.
(XLSX)

S2 Table List of licensing institutions. The sampling process was permitted by the museums or scientific institutions whose collection included the sampled anthropological material.
(XLSX)

S3 Table Interpretation. Interpretation of the strontium and oxygen stable isotope data.
(XLSX)

S1 Fig Boxplots of the ^87^Sr/^86^Sr ratios of females, males, undetermined adults, infants and adolescents per site and archaeological culture. Transdanubia (A), Croatia (C), and Alföld (C).
(TIF)

S2 Fig Boxplots of the ^87^Sr/^86^Sr ratios of females, males, undetermined adults, infants and adolescents per archaeological culture and site. Transdanubia and Croatia (A), Alföld (B).
(TIF)

S3 Fig Boxplots of the δ^18^O values of females, males, undetermined adults, infants and adolescents per site and archaeological culture. Transdanubia (A), Croatia (B), and Alföld (C).
(TIF)

S4 Fig Boxplots of the δ18O values of females, males, undetermined adults, infants and adolescents per archaeological culture and per site. Transdanubia and Croatia (A), Alföld (B).
(TIF)

S5 Fig ^87^Sr/^86^Sr ratios of each human dental enamel sample per site and archaeological culture from the Csabdi Télizöldes (CSAT), Bicske Galagonyás (BICS), Budakeszi 8. lh. Szőlőskert-Tangazdaság (BUD), and the Kóny 85 Enese (KON) sites. (A) Green lines represent the site-specific strontium isotope baseline range. The green dashed lines represent the baseline range that includes baseline outlier samples. (B) Light green lines represent the site-specific strontium isotope baseline range. The green dashed lines represent the baseline range that includes baseline outlier samples. Dark green lines represent the micro-regional baseline range that includes baseline samples from Budapest Békásmegyer [42]. (C) Green lines represent the site-specific strontium isotope baseline range. The green dashed lines represent the baseline range that includes baseline outlier samples.
(TIF)

S6 Fig ^87^Sr/^86^Sr ratios of each human dental enamel sample per site and archaeological culture from the Veszprém-Jutasi-Munkacsy út (VEJ), Felsőörs-Bárókert (FEB), Nemesvámos-Kapsa (NEK), Balatonszemes Bagódomb (BAB) and (Keszthely-Fenékpuszta Pusztaszentegyházi dűlő (KEFP). Light green lines represent the site-specific strontium isotope baseline range at each site. The green dashed lines represent the baseline range that includes baseline outlier samples at each site. Dark green lines represent the micro-regional baseline range that includes the baseline samples of these three sites.
(TIF)

S7 Fig ^87^Sr/^86^Sr ratios of each human dental enamel sample per site and archaeological culture from the Bölcske Gyürüsvölgy (BÖVÖ), Harta-Gátőrház (HARG), Fajsz (FAGA), Tolna-Mözs (TOLM), Lánycsók Gata Csatola (LGCS), Lanycsók Csata alja (M6-116), Szemely-Hegyes (SZEH), Szederkèny-Kukorica-dülö (SEKU), Borjád Kenderföldek (BORK) and Versend-Gilencsa (VEGI) sites. Light green lines represent the site-specific strontium isotope baseline range at each site. The green dashed lines represent the baseline range that includes baseline outlier samples at BÖVÖ. Dark green lines represent the micro-regional baseline range that includes the baseline samples of these four sites.
(TIF)

S8 Fig ^87^Sr/^86^Sr ratios of each human dental enamel sample per site and archaeological culture from the Vinkovci Jugobanka (VINJ), Vinkovci Nama (VINK), Vukovar Gimnazija (VUKG), Radovanci (RADO), Berettyóújfalu-nagy-Bócs dűlő (BENA), Ebes- Zsong-völgy (EBVÖ), Debrecen Tócópart Erdöalja (TOPE), Cegléd (CEG), Cegléd Ipari Park (CGIP), Tiszaföldvár Téglagyár (TITE), and Abony (ABO) sites. Light green lines represent the site-specific strontium isotope baseline range at each site. (A) Brown dots represent the additional ^87^Sr/^86^Sr ratios of human bones from the RADO, VINJ, VINK and VUKG sites. (B) Dark green lines represent the micro-regional baseline range suggested by C. Gerling for the same spatial area [36]. (C) Dashed green lines represent the baseline range that includes outlier baseline samples at ABO. Dark green lines represent the micro-regional baseline range at CEG and CGIP, which combines both site-specific baselines.
(TIF)

S9 Fig ^87^Sr/^86^Sr ratios of each human dental enamel sample per site and archaeological culture from Hódmezővásárhely-Gorzsa V. lh: Homokbànya (HOGO), Hódmezővásárhely Kotacpart (HOKO), HMV Kökénydomb (KÖKE), Deszk Ordos (DEOR), Szegvár Tűzköves (SZEG), Polgár-Piócási-Dűlő (POPI), Hejökürt-Lidl logisztikai központ (HELI), from the Tiszabura Bonishat (TIBO), Tiszaszőlős—Domaháza-puszta, Réti-dűlő (TIDO) and Pusztataksony Ledence (PULE) sites. (A and C) Green lines represent the site-specific and micro-regional strontium isotope baseline range for this site complex. (B) Light green lines represent the site-specific strontium isotope baseline range at each site. Dashed green lines represent the baseline range that includes outlier baseline samples at HELI. Dashed violet lines represent the micro-regional range at POPI. (C) Dashed green lines represent the baseline range that includes outlier baseline samples.
(TIF)

S10 Fig ^87^Sr/^86^Sr ratios of each human dental enamel sample per site and archaeological culture from the Tiszalök Hajnalos (TIHA), Mezőzombor–Községi temető (MEKÖ), Tiszadob-Okenéz (TISO), Hajdunanas-Eszlari ut (HAJE), Visonta (VISO), Kompolt-Kígyós-ér (KOKI), Mezökeresztes-Cethalom (MECE), Sajoszentpeter-vasúti örhaz (SAVÖ) and Garadna- Elkerülö (GAEL) sites. Light green lines represent the site-specific strontium isotope baseline range at each site. (A) Dashed green lines represent the baseline range that includes outlier baseline samples at MEKÖ. (B) Dashed green lines represent the baseline range that includes outlier baseline samples at MECE. Violet dashed lines represent at MECE the baseline range of Mezőkövesd-Moscolyas and at KOKI the baseline range of Füzesabony-Gubakut suggested by A. Whittle and colleagues [33]. (C) Dashed green lines represent the baseline range that includes outlier baseline samples at GAEL. No strontium isotope baseline could be determined at SAVÖ.
(TIF)

S11 Fig δ^18^O value of each human dental enamel sample from the Kóny 85 Enese (KON), Budakeszi 8. lh. Szőlőskert-Tangazdaság (BUD), Csabdi Télizöldes (CSAT), Bölcske Gyürüsvölgy M3-TO 14. lh. (BÖVÖ), Tolna-Mözs (TOLM), Felsőörs-Bárókert (FEB), Alsónyék elkerülő (ALE), Bátaszék-Lajvér (BAL) and Bátaszék-Mérnöki telep (BAM) sites. The green dashed lines represent the local oxygen isotope baseline range calculated from the long-term annual average δ^18^O value of modern precipitation data [37–40] from each site ± 1 ‰. The blue dashed line represents the average δ^18^O value of the modern river Danube in Hungary [66].
(TIF)

S12 Fig δ^18^O value of each human dental enamel sample from the Mórágy Tűzkődomb (MORT), Lánycsók Gata Csatola (LGCS), Lanycsók Csata alja (M6), Versend-Gilencsa (VEGI), Vinkovci Jugobanka (VINJ), Vinkovci Nama (VINK), Vukovar Gimnazija (VUKG), Vela Spila, Korčula (STANKO), Hódmezővásárhely Kotacpart (HOKO), Berettyóújfalu-nagy-Bócs dűlő (BENA), Ebes- Zsong-völgy (EBVÖ), Debrecen Tócópart Erdöalja (TOPE), Tiszaföldvár Téglagyár (TITE), Cegléd Ipari Park (CGIP), Polgár-Piócási-Dűlő (POPI), Sajoszentpeter-vasúti örhaz (SAVÖ), Tiszabura Bonishat (TIBO), Tiszaszőlős—Domaháza-puszta, Réti-dűlő (TIDO) and Tiszalök Hajnalos (TIHA) sites. The green dashed lines represent the local oxygen isotope baseline range calculated from the long-term annual average δ^18^O value of modern precipitation data [37–40] from each site ± 1 ‰. The violet dashed lines represent the baseline range suggested by C. Gerling [36] in the spatial area around BENA, EBVÖ, and TOPE.
(TIF)

S13 Fig δ^18^O and ^87^Sr/^86^Sr data of each sample and individual. (A) At the Kóny 85 Enese (KON) site. The green lines represent the local/site-specific oxygen and isotope baseline at the site. Samples from a same individual are related by a grey line. (B) At the Tolna-Mözs (TOLM) site. The green lines represent the local/site-specific oxygen and isotope baseline at the site. Samples from a same individual are related by a grey line. (C) At the Csabdi Télizöldes (CSAT) site. The green lines represent the local/site-specific oxygen and isotope baseline at the site. Samples from a same individual are related by a grey line.
(TIF)

S14 Fig δ^18^O and ^87^Sr/^86^Sr data of each sample and individual. (A) At the Budakeszi 8. lh. Szőlőskert-Tangazdaság (BUD) site. The green lines represent the local/site-specific oxygen and isotope baseline at the site. The violet line represents the extension of the micro-regional strontium isotope baseline when including the baseline samples from Budapest Békásmegyer [41]. Samples from a same individual are related by a grey line. (B) At the Bölcske Gyürüsvölgy M3-TO 14. lh. (BÖVÖ) site. The green lines represent the local/site-specific oxygen and isotope baseline at the site. Samples from a same individual are related by a grey line. (C) At the Felsőörs-Bárókert (FEB) site. The green lines represent the local/site-specific oxygen and isotope baseline at the site. Samples from a same individual are related by a grey line.
(TIF)

S15 Fig δ^18^O and ^87^Sr/^86^Sr data of each sample and individual. (A) At the Alsónyék elkerülő (ALE) site. (B) At the Bátaszék-Lajvér (BAL) site. (C) At the Bátaszék-Mérnöki telep (BAM) site. The light green lines represent the site-specific isotope baseline ranges at each site. The dark green lines represent the micro-regional baseline range. Samples from a same individual are related by a grey line.
(TIF)

S16 Fig δ^18^O and ^87^Sr/^86^Sr data of each sample and individual. (A) the Mórágy Tűzkődomb (MORT) site. The green lines represent the site-specific/local oxygen and strontium isotope baseline ranges. (B) At the Lanycsók Csata alja (M6-116) site. The light green lines represent the site-specific/local oxygen and strontium isotope baseline ranges. The dark green lines represent the micro-regional strontium baseline range. (C) At the Versend-Gilencsa (VEGI) site. The light green lines represent the site-specific/local oxygen and strontium isotope baseline ranges. The dark green lines represent the micro-regional strontium baseline range.
(TIF)

S17 Fig δ^18^O and ^87^Sr/^86^Sr data of each Croatian sample and individual. Vinkovci Jugobanka (VINJ), Vinkovci Nama (VINK), and Vukovar Gimnazija (VUKG). The green lines represent the site-specific/ oxygen and strontium isotope baseline ranges.
(TIF)

S18 Fig δ^18^O and ^87^Sr/^86^Sr data of each sample and individual. (A) At the Hódmezővásárhely Kotacpart (HOKO) site. The green lines represent the site-specific/local oxygen and strontium isotope baseline ranges. (B) From the Tiszalök Hajnalos (TIHA) and the Sajoszentpeter-vasúti örhaz (SAVÖ) sites. No strontium isotope baseline could be determined at TIHA and at SAVÖ. The oxygen isotope baseline range (15,57 to 17,57 ‰) is not represented on the diagrams.
(TIF)

S19 Fig δ^18^O and ^87^Sr/^86^Sr data of each sample and individual. (A) At the Cegléd Ipari Park (CGIP) site. The light green lines represent the site-specific/local oxygen and strontium isotope baseline ranges. The dark green lines represent the micro-regional strontium isotope baseline range. (B) the Berettyóújfalu-nagy-Bócs dűlő (BENA) site. The green lines represent the site-specific/local oxygen and strontium isotope baseline ranges. The violet lines represent the baseline range suggested by C. Gerling [36] in the same spatial area. (C) At the Ebes-Zsong-völgy (EBVÖ) and Debrecen Tócópart Erdöalja (TOPE) sites. The green lines represent the site-specific/local oxygen and strontium isotope baseline ranges at the EBVÖ site. The orange lines represent the site-specific/local oxygen and strontium isotope baseline ranges at the TOPE site.
(TIF)

S20 Fig δ^18^O and ^87^Sr/^86^Sr data of each sample and individual. (A) At the Polgár-Piócási-Dűlő (POPI) site. The light green lines represent the local oxygen and strontium isotope baseline ranges. The violet lines represent the micro-regional strontium isotope baseline range. (B) At the Tiszabura Bonishat (TIBO) and Tiszaszőlős-Domaháza-puszta, Réti-dűlő (TIDO) sites. The light green lines represent the local oxygen and strontium isotope baseline ranges. (C) Tiszaföldvár Téglagyár (TITE) site. The green lines represent the site-specific/local oxygen and strontium isotope baseline ranges.
(TIF)

S21 Fig Proportion of site-specific, mobile, micro-regional and non-local individuals at each site per archaeological culture. (A) Starčevo and Körös. (B) TLBK, ALBK, and Vinča. (C) Sopot, Esztár, Tiszadob/Bükk, and Szakálhát. (D) Lengyel and Tisza. (E) Balaton-Lasinja and Tiszapolgár.
(PDF)
