# Supplementary figures and images for: Tracing mobility patterns through the 6th-5th millennia BC in the Carpathian Basin with strontium and oxygen stable isotope analyses
Source: PLoS One. 2020 Dec 9;15(12):e0242745. doi: 10.1371/journal.pone.0242745 (PMC7725410; doi:10.1371/journal.pone.0242745)

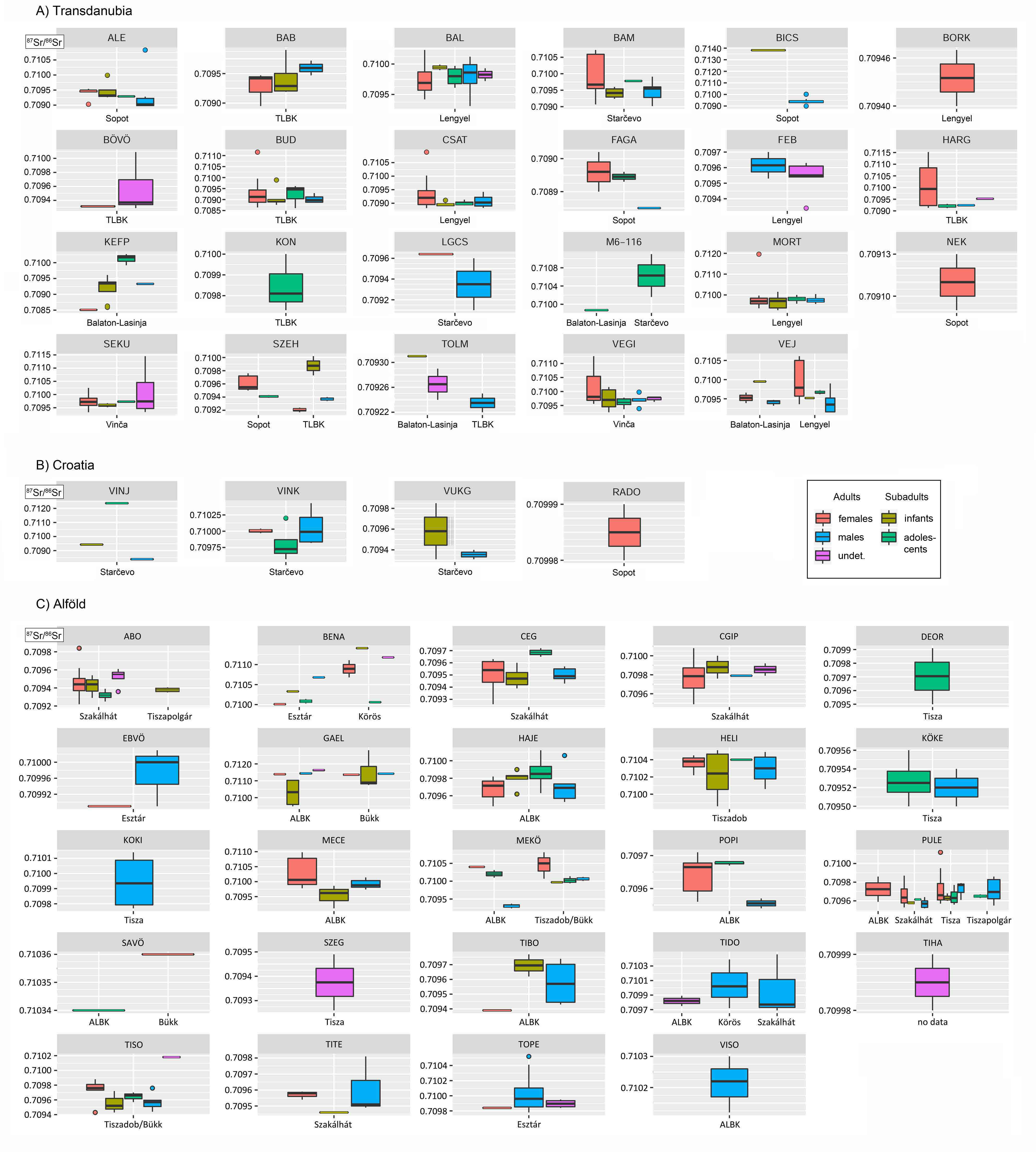

Supplement: S1 Fig — Transdanubia (A), Croatia (C), and Alföld (C). (TIF) [file pone.0242745.s004.tif]

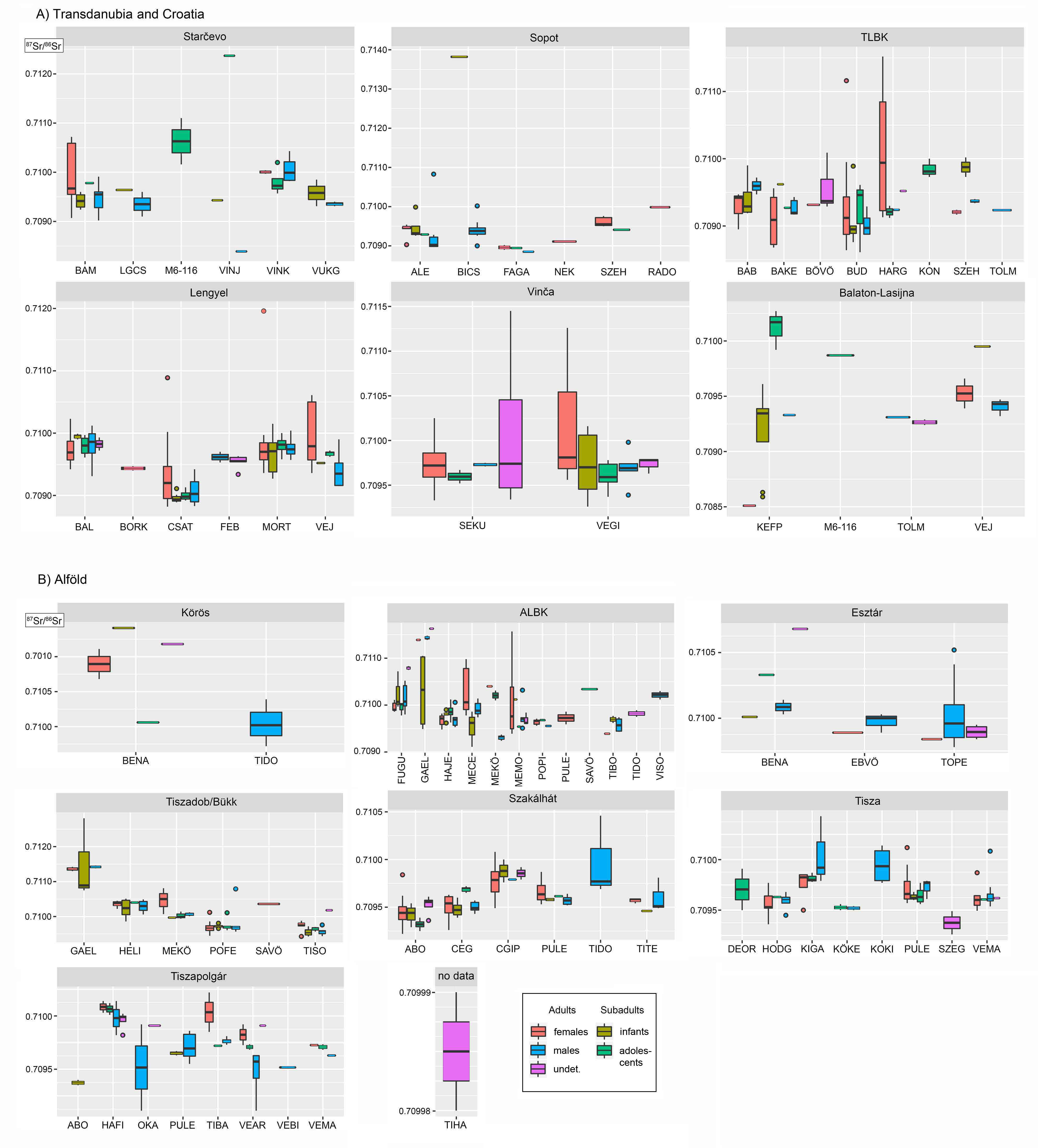

Supplement: S2 Fig — Transdanubia and Croatia (A), Alföld (B). (TIF) [file pone.0242745.s005.tif]

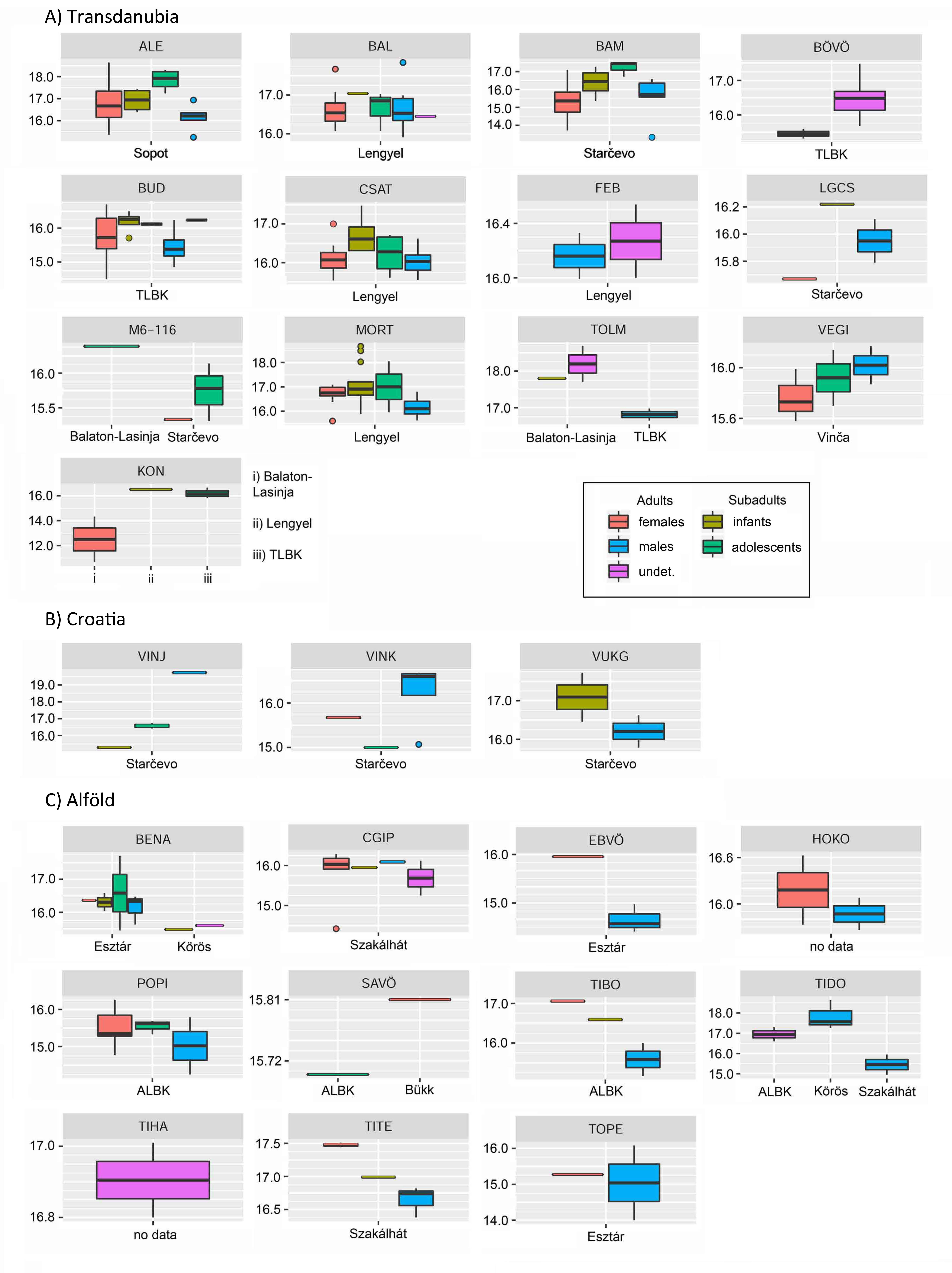

Supplement: S3 Fig — Transdanubia (A), Croatia (B), and Alföld (C). (TIF) [file pone.0242745.s006.tif]

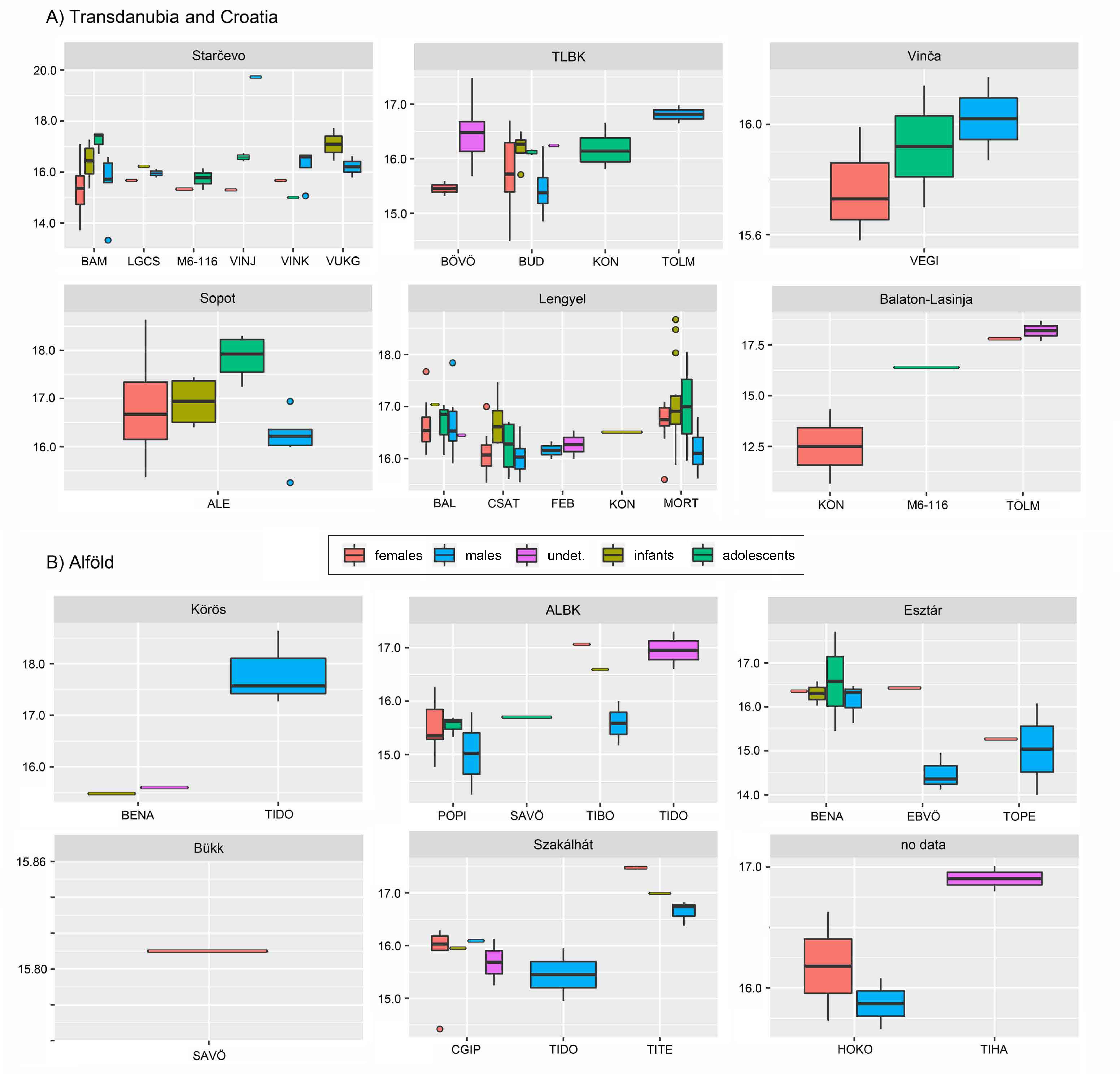

Supplement: S4 Fig — Transdanubia and Croatia (A), Alföld (B). (TIF) [file pone.0242745.s007.tif]

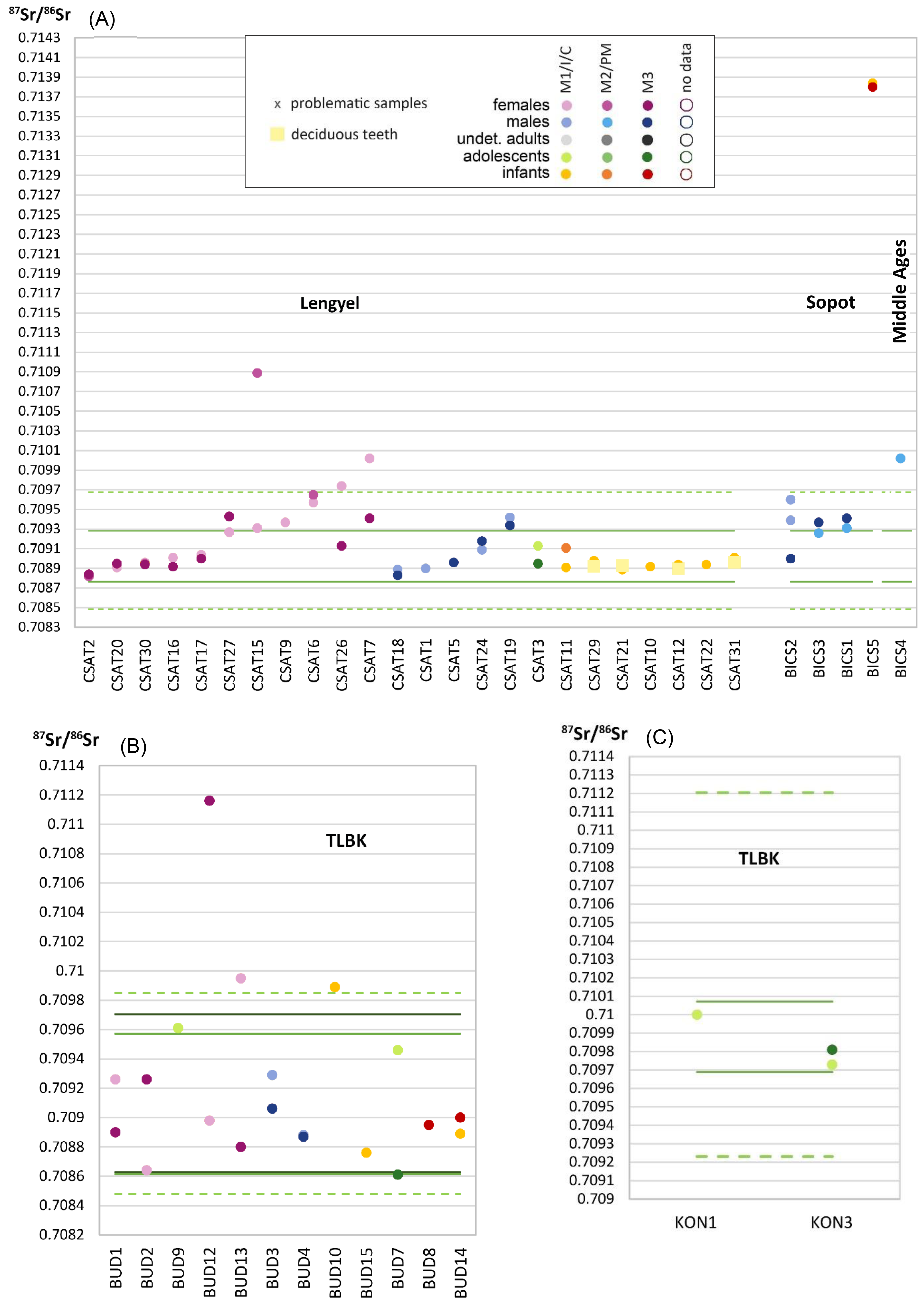

Supplement: S5 Fig — (A) Green lines represent the site-specific strontium isotope baseline range. The green dashed lines represent the baseline range that includes baseline outlier samples. (B) Light green lines represent the site-specific strontium isotope baseline range. The green dashed lines represent the baseline range that includes baseline outlier samples. Dark green lines represent the micro-regional baseline range that includes baseline samples from Budapest Békásmegyer [42]. (C) Green lines represent the site-specific strontium isotope baseline range. The green dashed lines represent the baseline range that includes baseline outlier samples. (TIF) [file pone.0242745.s008.tif]

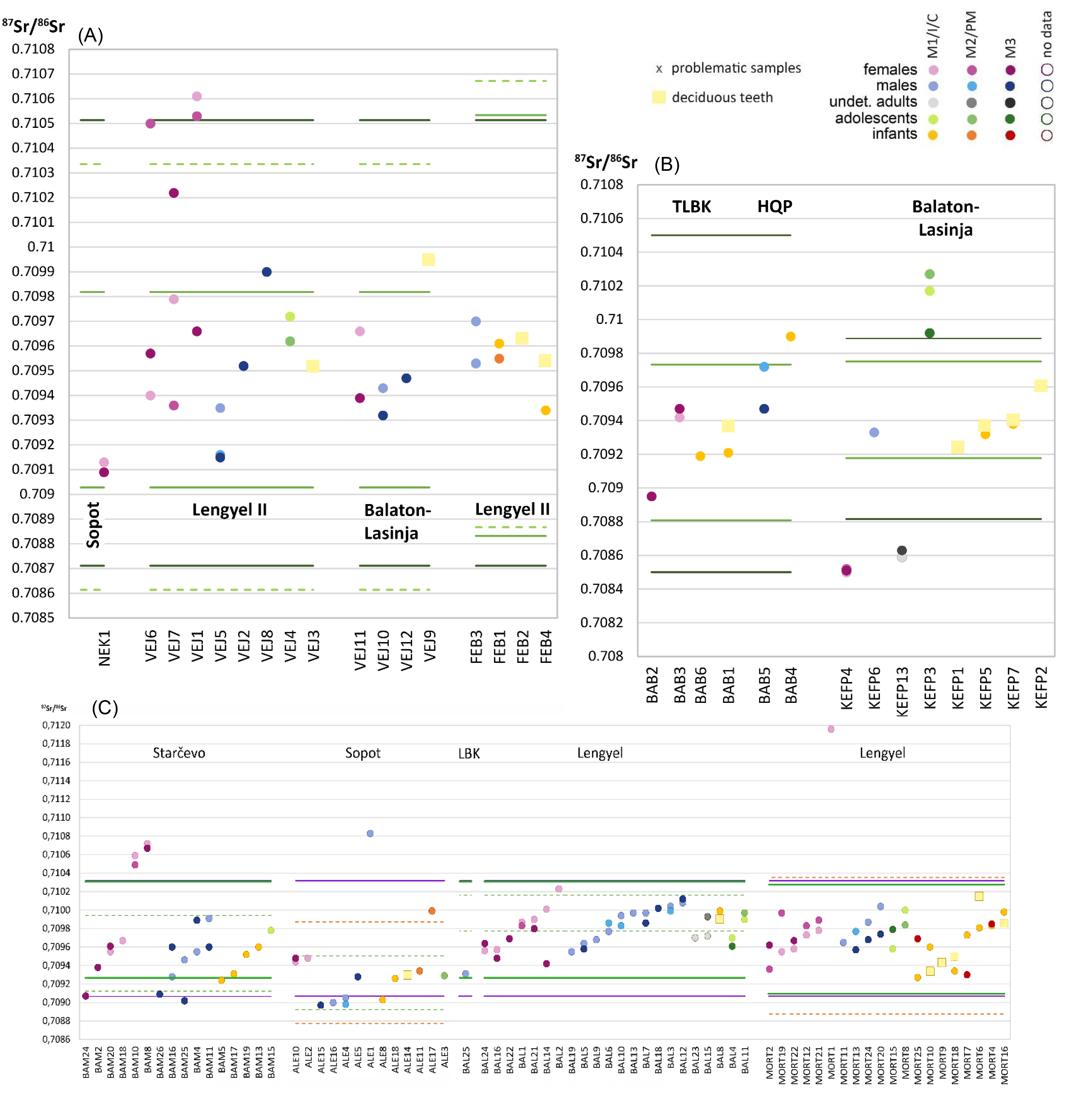

Supplement: S6 Fig — Light green lines represent the site-specific strontium isotope baseline range at each site. The green dashed lines represent the baseline range that includes baseline outlier samples at each site. Dark green lines represent the micro-regional baseline range that includes the baseline samples of these three sites. (TIF) [file pone.0242745.s009.tif]

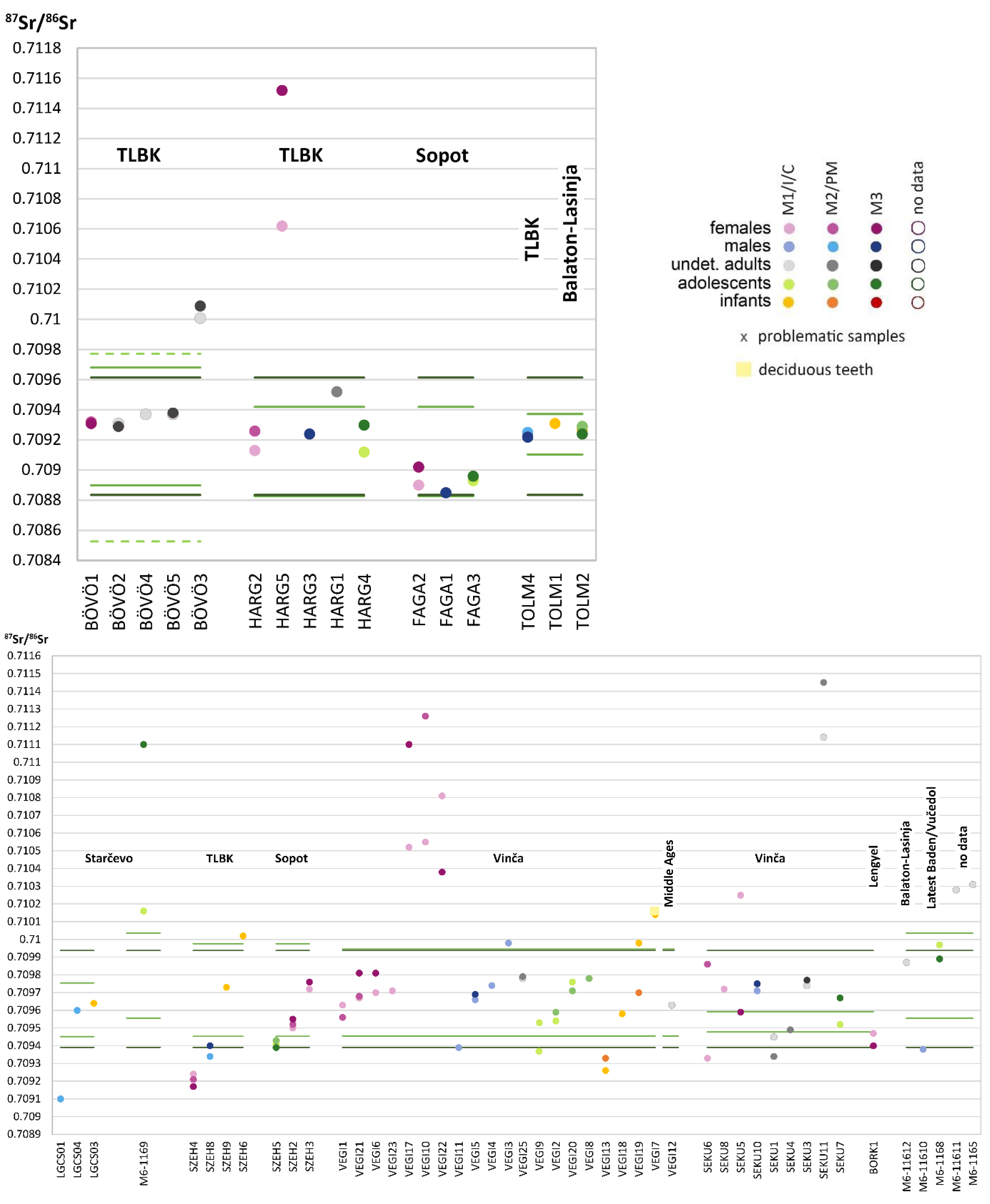

Supplement: S7 Fig — Light green lines represent the site-specific strontium isotope baseline range at each site. The green dashed lines represent the baseline range that includes baseline outlier samples at BÖVÖ. Dark green lines represent the micro-regional baseline range that includes the baseline samples of these four sites. (TIF) [file pone.0242745.s010.tif]

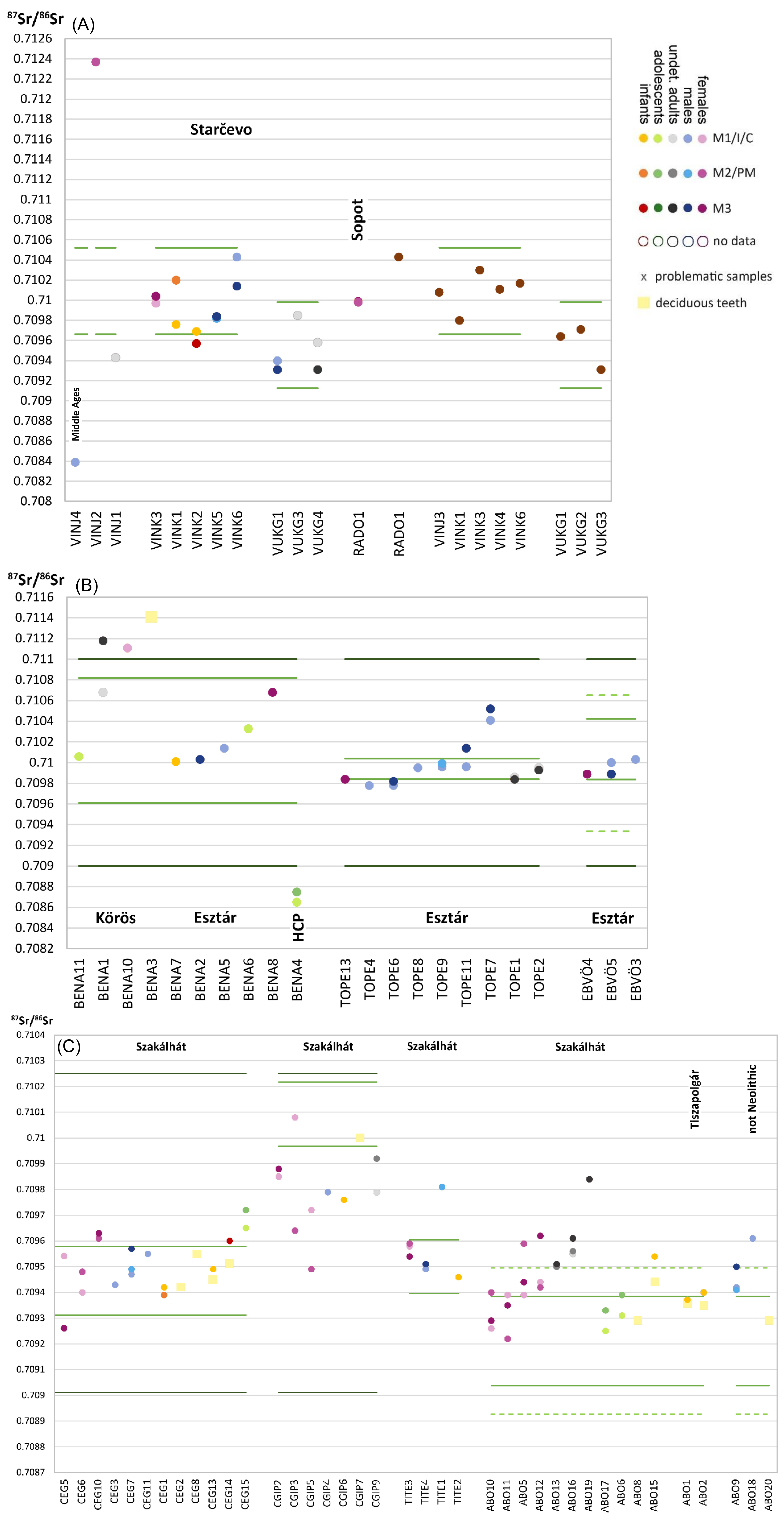

Supplement: S8 Fig — Light green lines represent the site-specific strontium isotope baseline range at each site. (A) Brown dots represent the additional 87Sr/86Sr ratios of human bones from the RADO, VINJ, VINK and VUKG sites. (B) Dark green lines represent the micro-regional baseline range suggested by C. Gerling for the same spatial area [36]. (C) Dashed green lines represent the baseline range that includes outlier baseline samples at ABO. Dark green lines represent the micro-regional baseline range at CEG and CGIP, which combines both site-specific baselines. (TIF) [file pone.0242745.s011.tif]

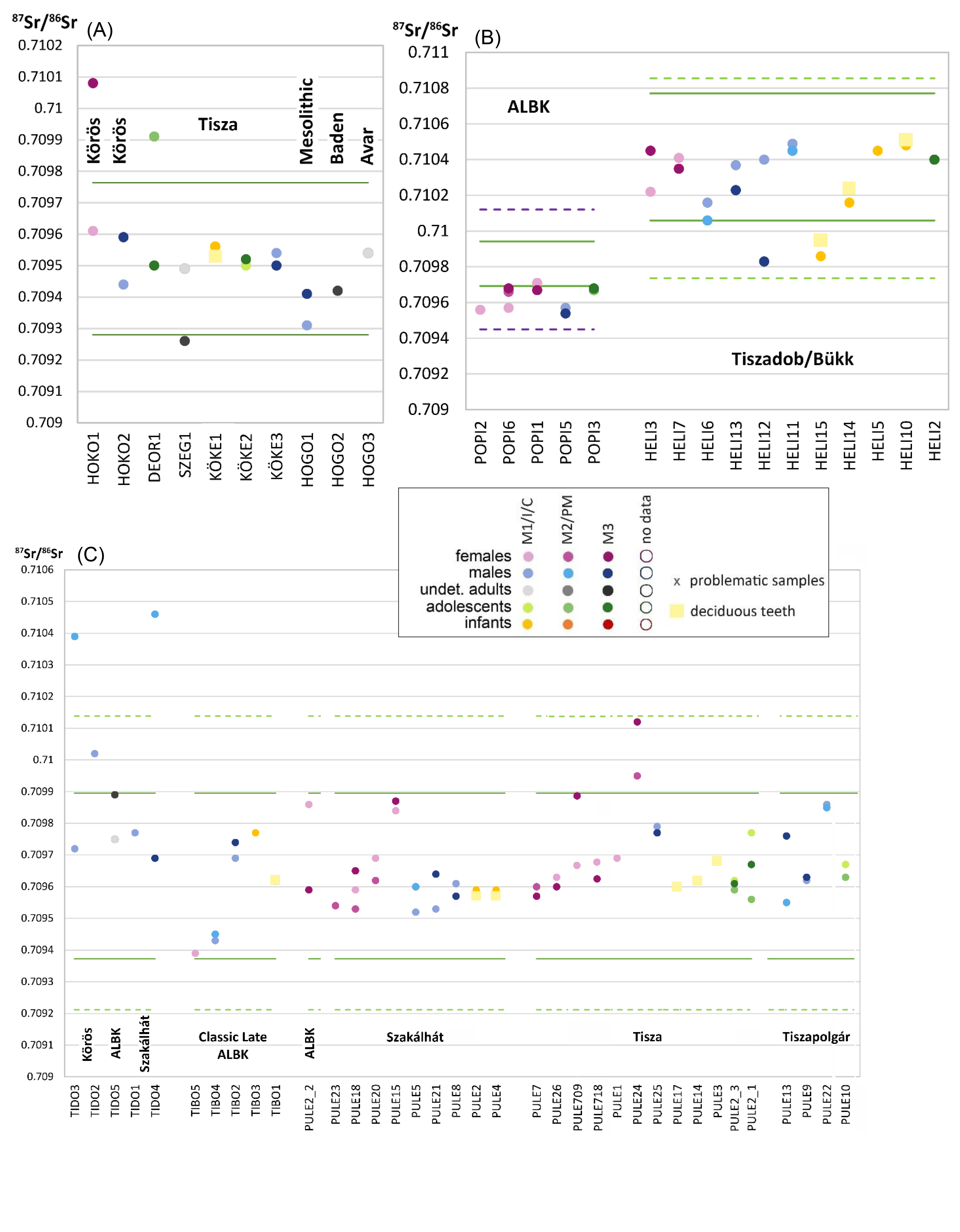

Supplement: S9 Fig — (A and C) Green lines represent the site-specific and micro-regional strontium isotope baseline range for this site complex. (B) Light green lines represent the site-specific strontium isotope baseline range at each site. Dashed green lines represent the baseline range that includes outlier baseline samples at HELI. Dashed violet lines represent the micro-regional range at POPI. (C) Dashed green lines represent the baseline range that includes outlier baseline samples. (TIF) [file pone.0242745.s012.tif]

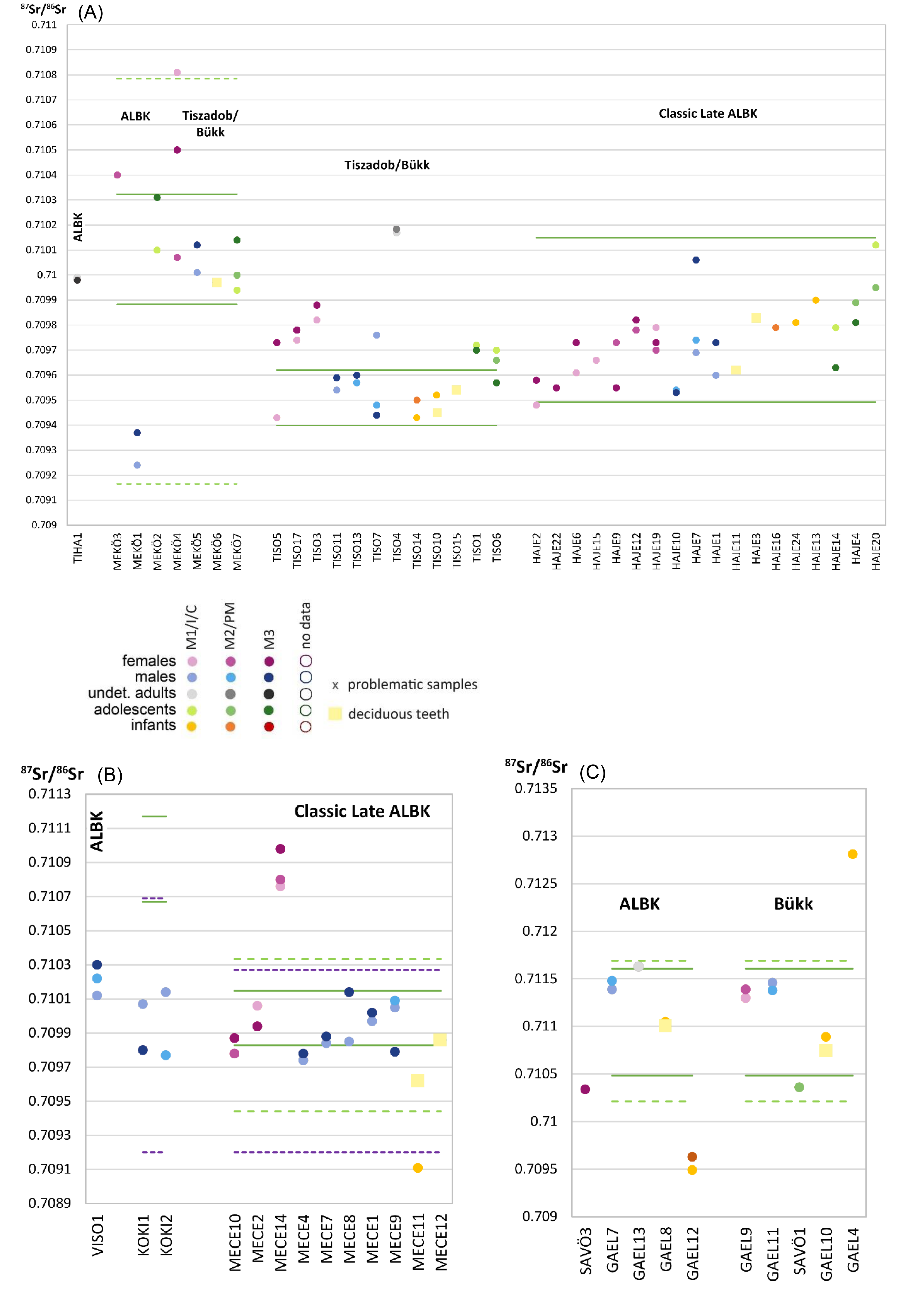

Supplement: S10 Fig — Light green lines represent the site-specific strontium isotope baseline range at each site. (A) Dashed green lines represent the baseline range that includes outlier baseline samples at MEKÖ. (B) Dashed green lines represent the baseline range that includes outlier baseline samples at MECE. Violet dashed lines represent at MECE the baseline range of Mezőkövesd-Moscolyas and at KOKI the baseline range of Füzesabony-Gubakut suggested by A. Whittle and colleagues [33]. (C) Dashed green lines represent the baseline range that includes outlier baseline samples at GAEL. No strontium isotope baseline could be determined at SAVÖ. (TIF) [file pone.0242745.s013.tif]

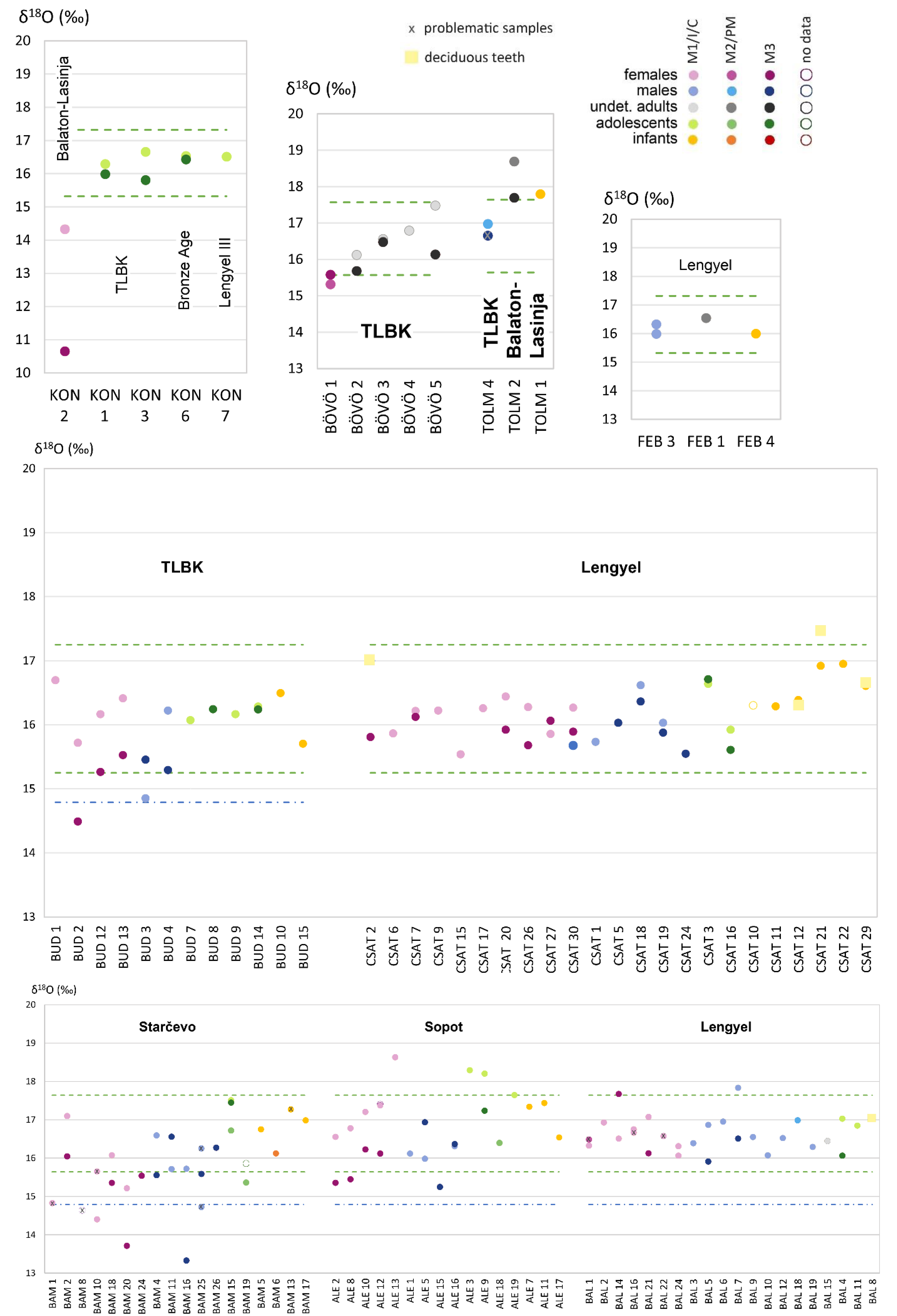

Supplement: S11 Fig — The green dashed lines represent the local oxygen isotope baseline range calculated from the long-term annual average δ18O value of modern precipitation data [37–40] from each site ± 1 ‰. The blue dashed line represents the average δ18O value of the modern river Danube in Hungary [66]. (TIF) [file pone.0242745.s014.tif]

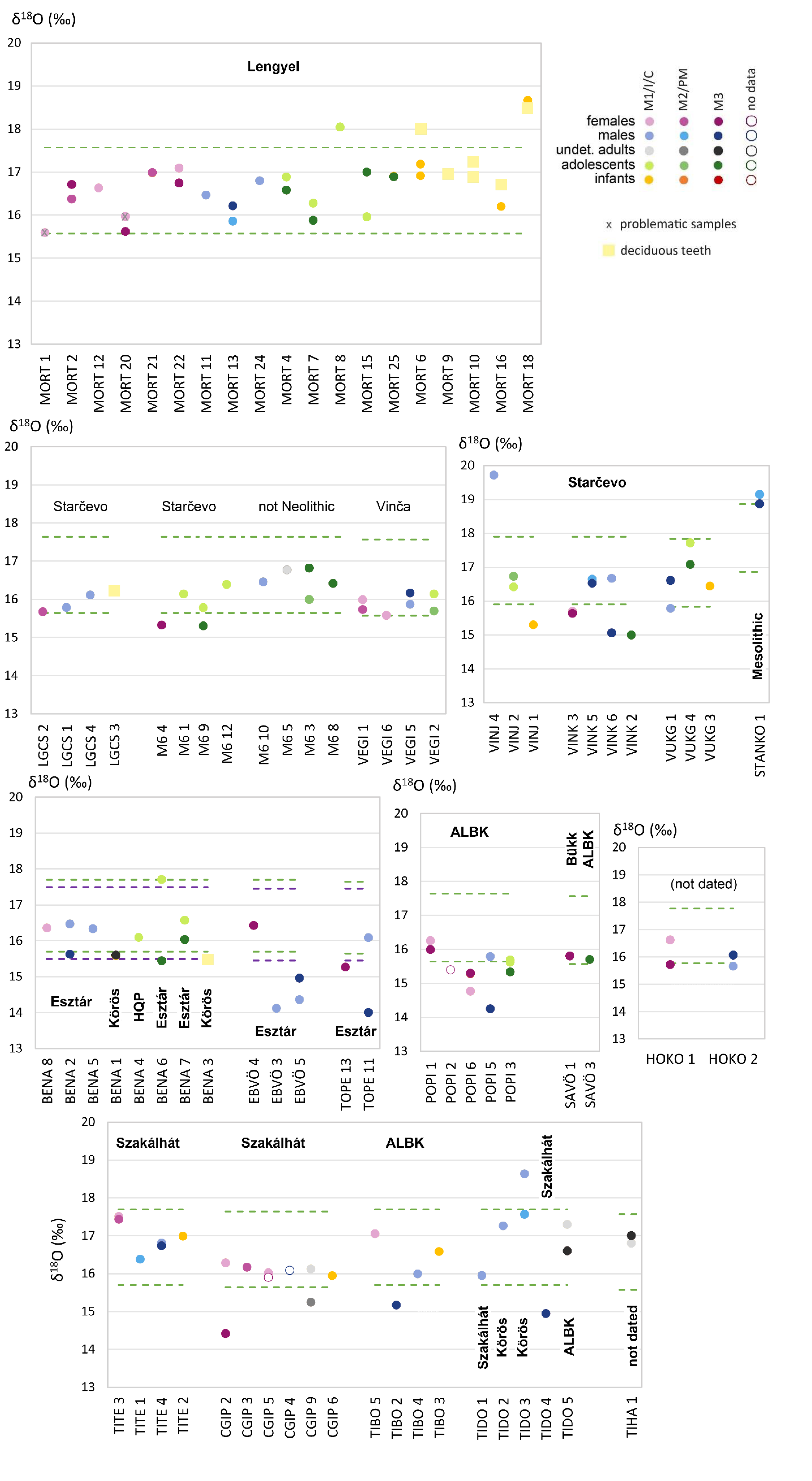

Supplement: S12 Fig — The green dashed lines represent the local oxygen isotope baseline range calculated from the long-term annual average δ18O value of modern precipitation data [37–40] from each site ± 1 ‰. The violet dashed lines represent the baseline range suggested by C. Gerling [36] in the spatial area around BENA, EBVÖ, and TOPE. (TIF) [file pone.0242745.s015.tif]

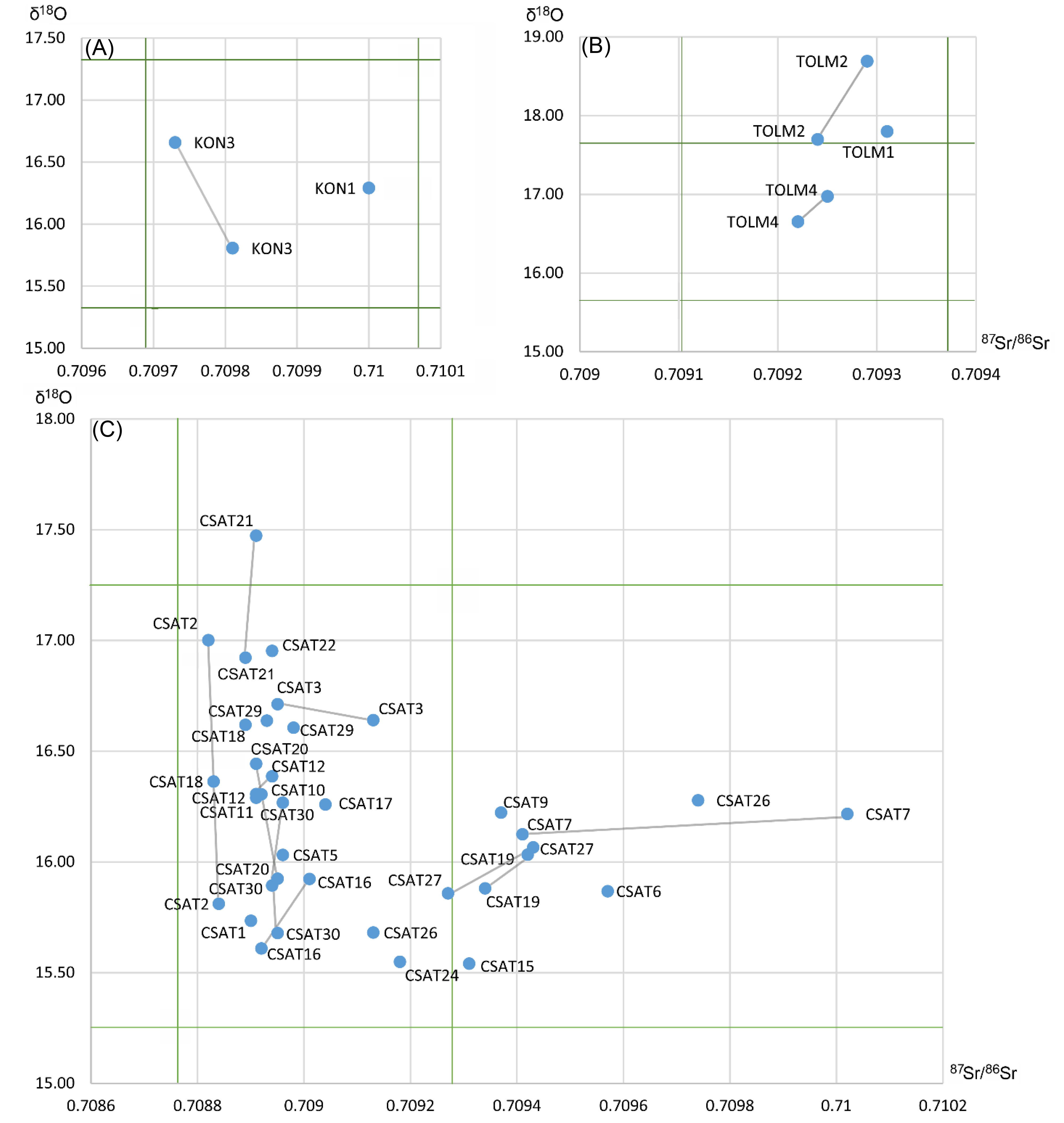

Supplement: S13 Fig — (A) At the Kóny 85 Enese (KON) site. The green lines represent the local/site-specific oxygen and isotope baseline at the site. Samples from a same individual are related by a grey line. (B) At the Tolna-Mözs (TOLM) site. The green lines represent the local/site-specific oxygen and isotope baseline at the site. Samples from a same individual are related by a grey line. (C) At the Csabdi Télizöldes (CSAT) site. The green lines represent the local/site-specific oxygen and isotope baseline at the site. Samples from a same individual are related by a grey line. (TIF) [file pone.0242745.s016.tif]

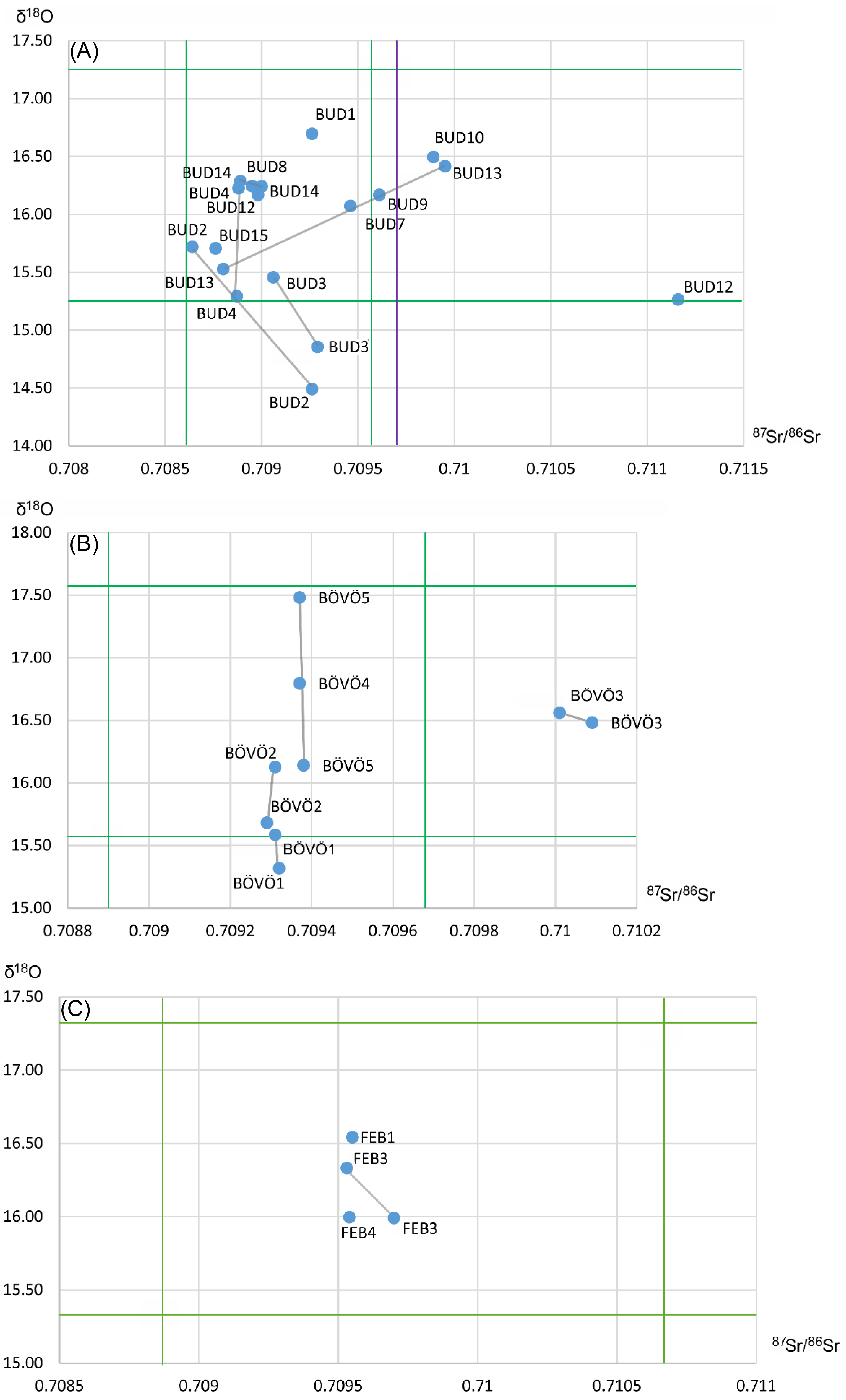

Supplement: S14 Fig — (A) At the Budakeszi 8. lh. Szőlőskert-Tangazdaság (BUD) site. The green lines represent the local/site-specific oxygen and isotope baseline at the site. The violet line represents the extension of the micro-regional strontium isotope baseline when including the baseline samples from Budapest Békásmegyer [41]. Samples from a same individual are related by a grey line. (B) At the Bölcske Gyürüsvölgy M3-TO 14. lh. (BÖVÖ) site. The green lines represent the local/site-specific oxygen and isotope baseline at the site. Samples from a same individual are related by a grey line. (C) At the Felsőörs-Bárókert (FEB) site. The green lines represent the local/site-specific oxygen and isotope baseline at the site. Samples from a same individual are related by a grey line. (TIF) [file pone.0242745.s017.tif]

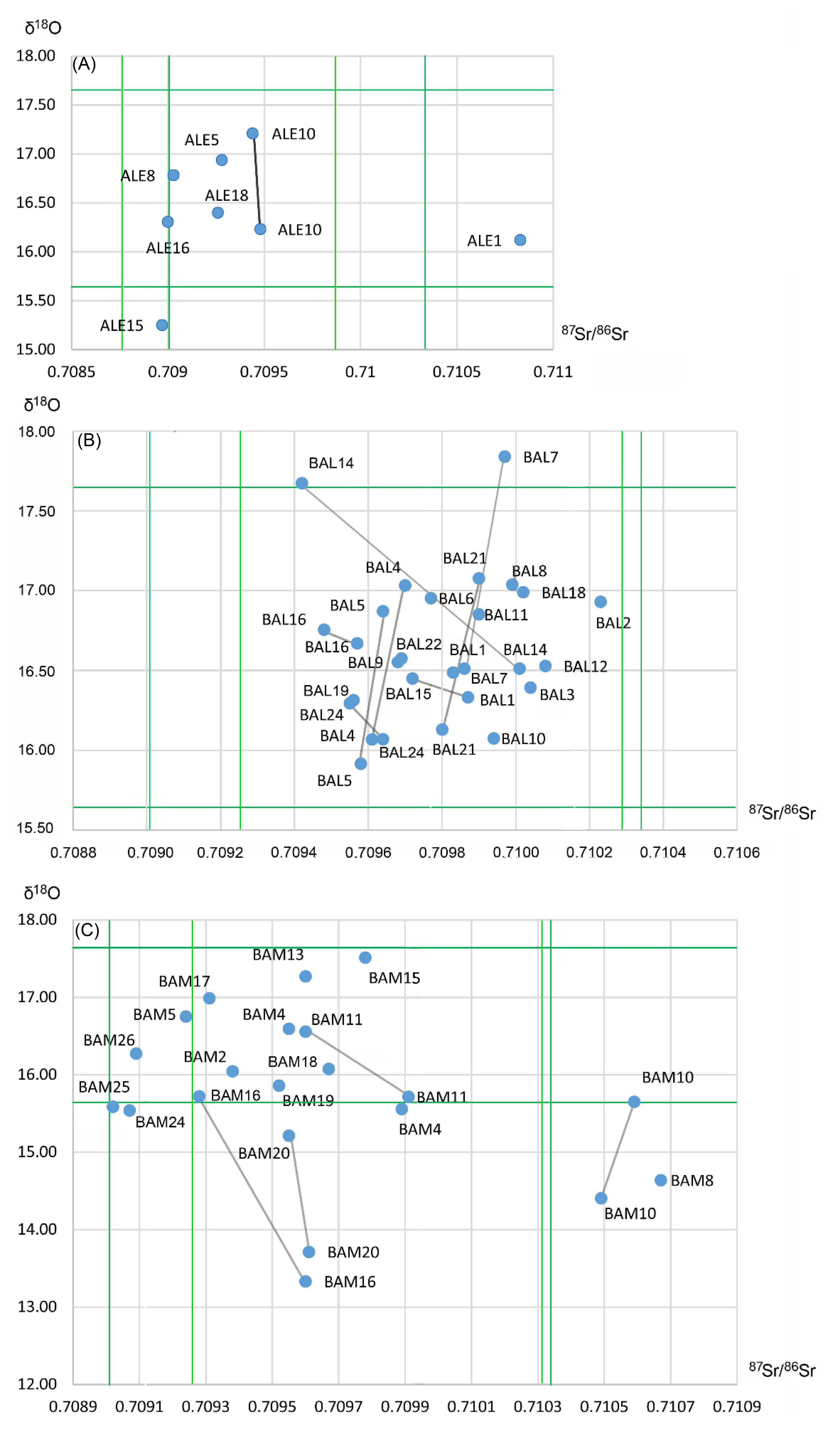

Supplement: S15 Fig — (A) At the Alsónyék elkerülő (ALE) site. (B) At the Bátaszék-Lajvér (BAL) site. (C) At the Bátaszék-Mérnöki telep (BAM) site. The light green lines represent the site-specific isotope baseline ranges at each site. The dark green lines represent the micro-regional baseline range. Samples from a same individual are related by a grey line. (TIF) [file pone.0242745.s018.tif]

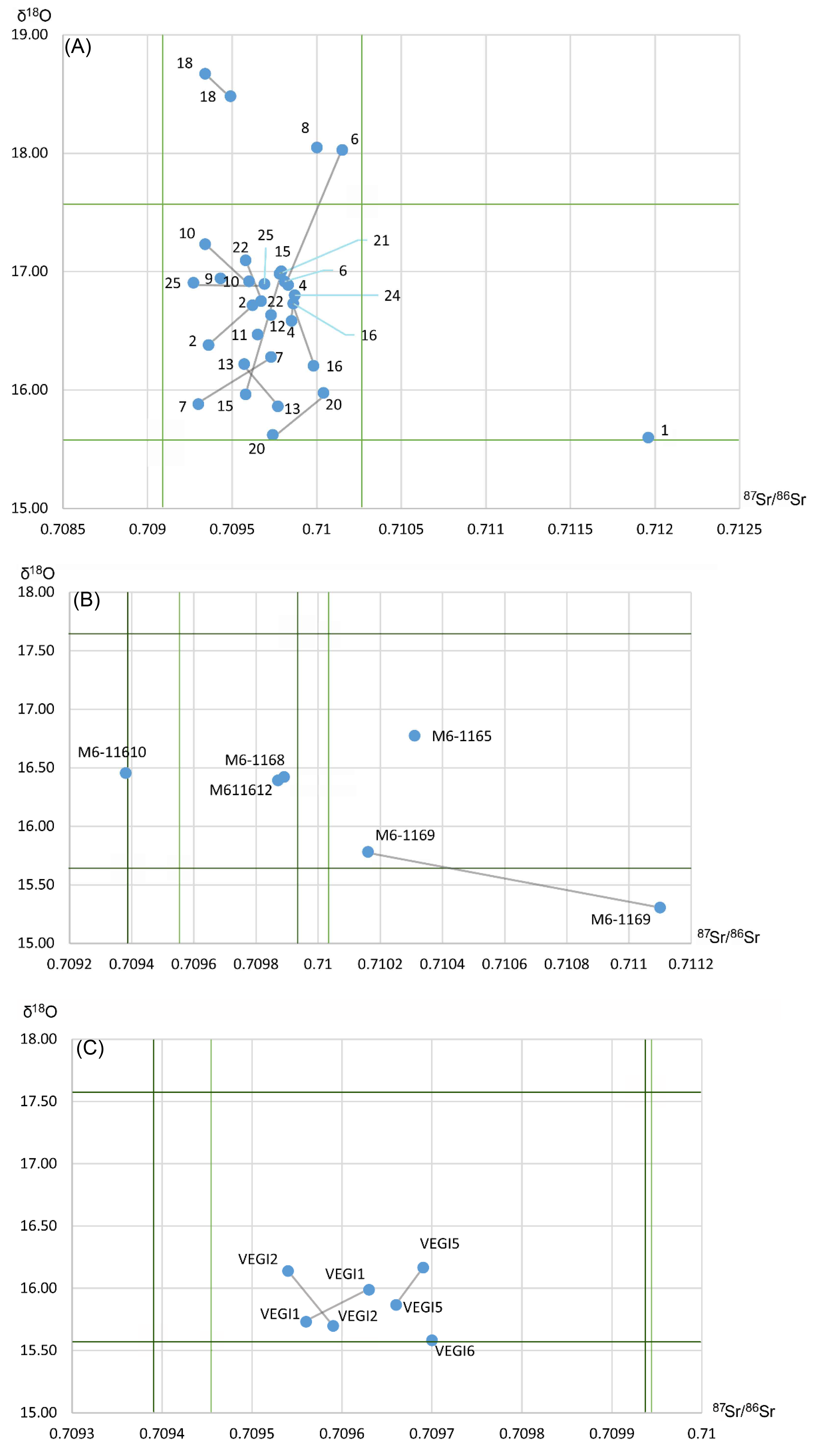

Supplement: S16 Fig — (A) the Mórágy Tűzkődomb (MORT) site. The green lines represent the site-specific/local oxygen and strontium isotope baseline ranges. (B) At the Lanycsók Csata alja (M6-116) site. The light green lines represent the site-specific/local oxygen and strontium isotope baseline ranges. The dark green lines represent the micro-regional strontium baseline range. (C) At the Versend-Gilencsa (VEGI) site. The light green lines represent the site-specific/local oxygen and strontium isotope baseline ranges. The dark green lines represent the micro-regional strontium baseline range. (TIF) [file pone.0242745.s019.tif]

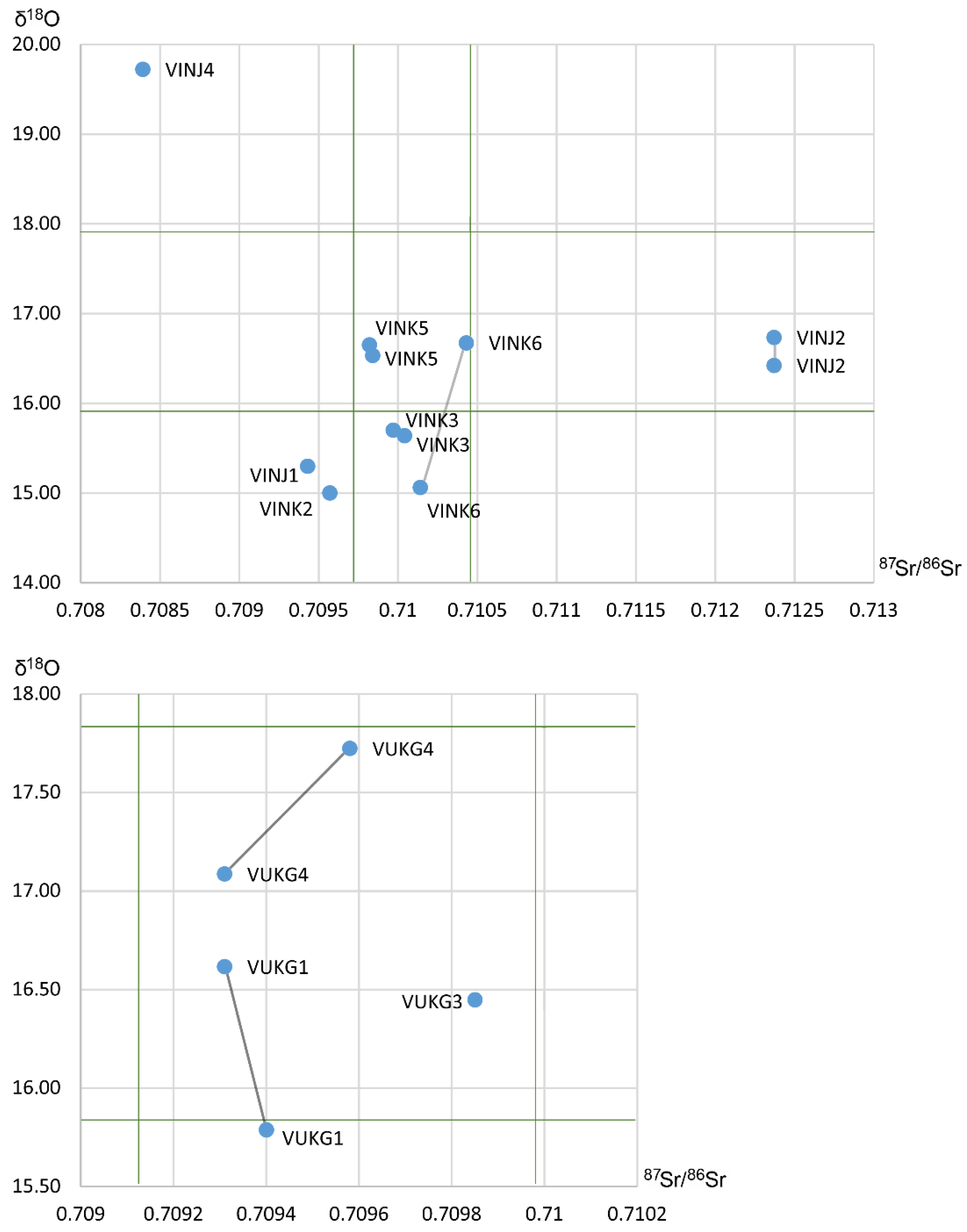

Supplement: S17 Fig — Vinkovci Jugobanka (VINJ), Vinkovci Nama (VINK), and Vukovar Gimnazija (VUKG). The green lines represent the site-specific/ oxygen and strontium isotope baseline ranges. (TIF) [file pone.0242745.s020.tif]

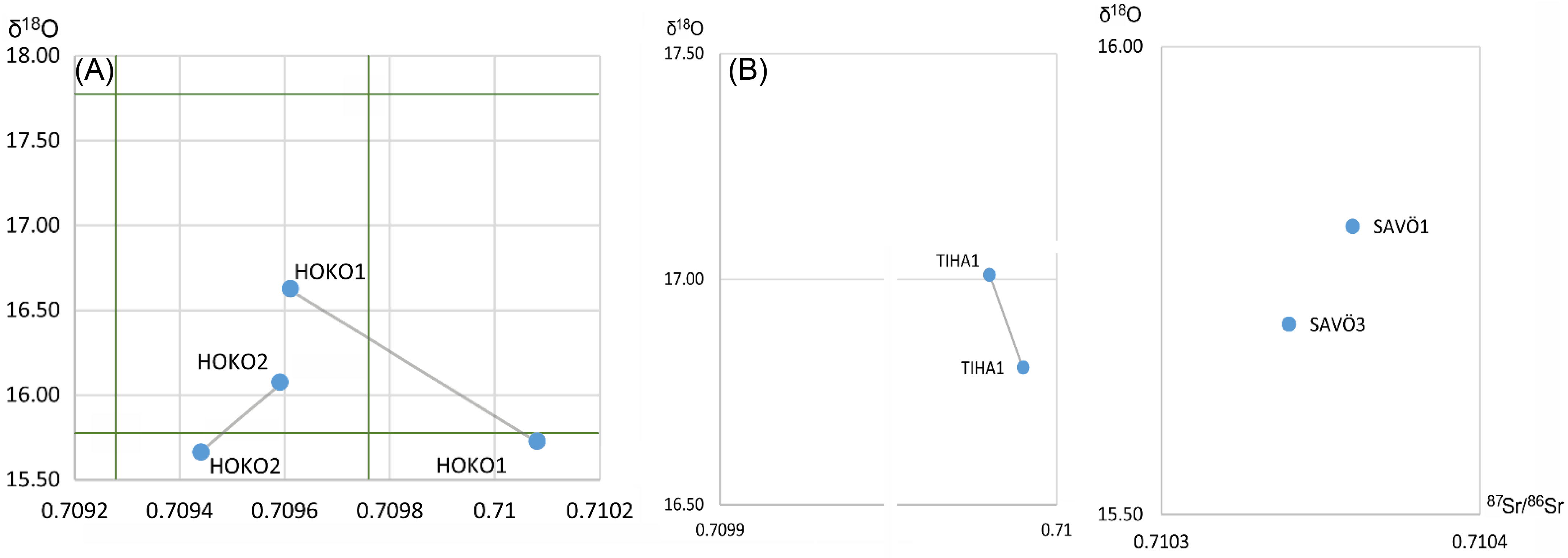

Supplement: S18 Fig — (A) At the Hódmezővásárhely Kotacpart (HOKO) site. The green lines represent the site-specific/local oxygen and strontium isotope baseline ranges. (B) From the Tiszalök Hajnalos (TIHA) and the Sajoszentpeter-vasúti örhaz (SAVÖ) sites. No strontium isotope baseline could be determined at TIHA and at SAVÖ. The oxygen isotope baseline range (15,57 to 17,57 ‰) is not represented on the diagrams. (TIF) [file pone.0242745.s021.tif]

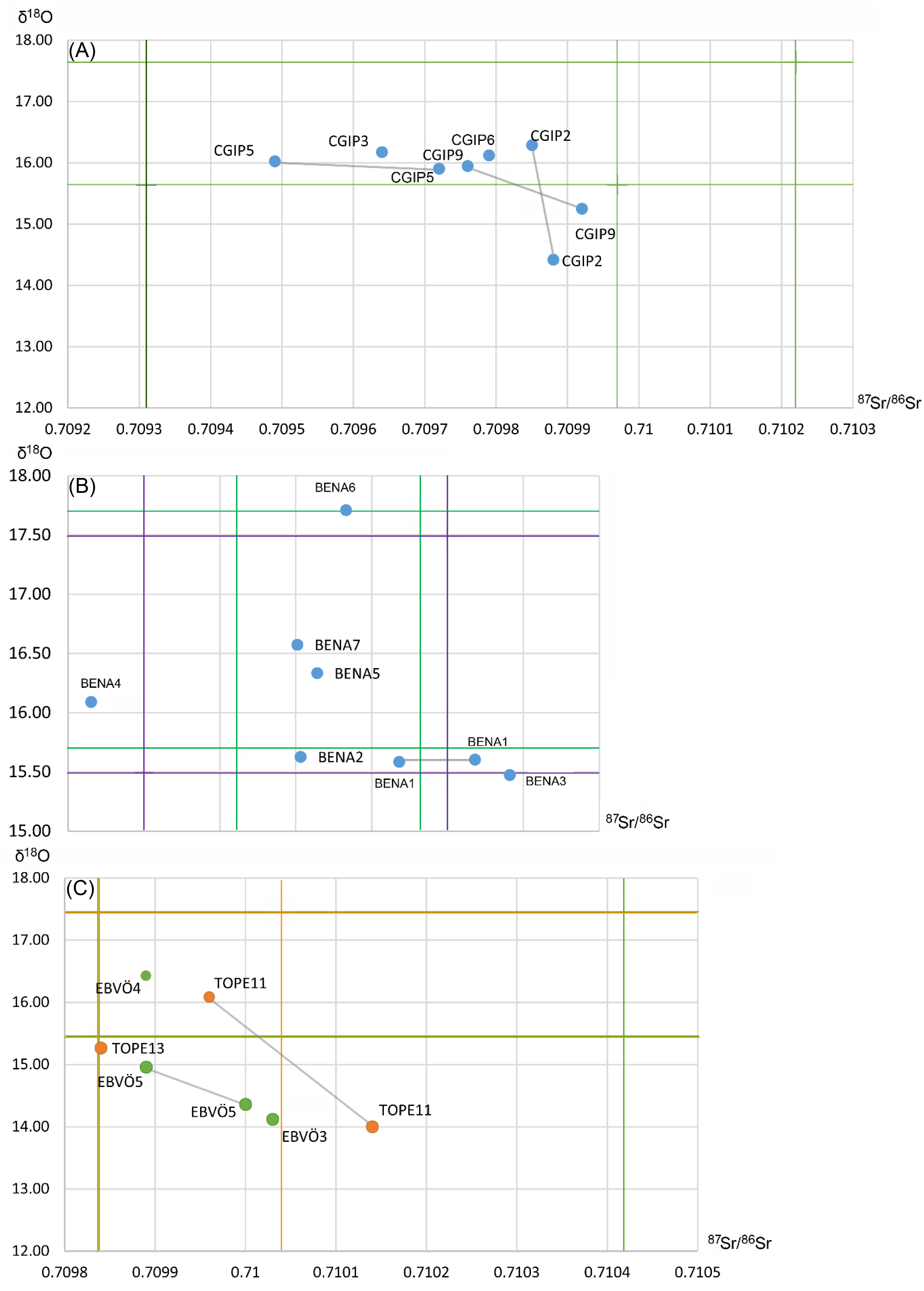

Supplement: S19 Fig — (A) At the Cegléd Ipari Park (CGIP) site. The light green lines represent the site-specific/local oxygen and strontium isotope baseline ranges. The dark green lines represent the micro-regional strontium isotope baseline range. (B) the Berettyóújfalu-nagy-Bócs dűlő (BENA) site. The green lines represent the site-specific/local oxygen and strontium isotope baseline ranges. The violet lines represent the baseline range suggested by C. Gerling [36] in the same spatial area. (C) At the Ebes-Zsong-völgy (EBVÖ) and Debrecen Tócópart Erdöalja (TOPE) sites. The green lines represent the site-specific/local oxygen and strontium isotope baseline ranges at the EBVÖ site. The orange lines represent the site-specific/local oxygen and strontium isotope baseline ranges at the TOPE site. (TIF) [file pone.0242745.s022.tif]

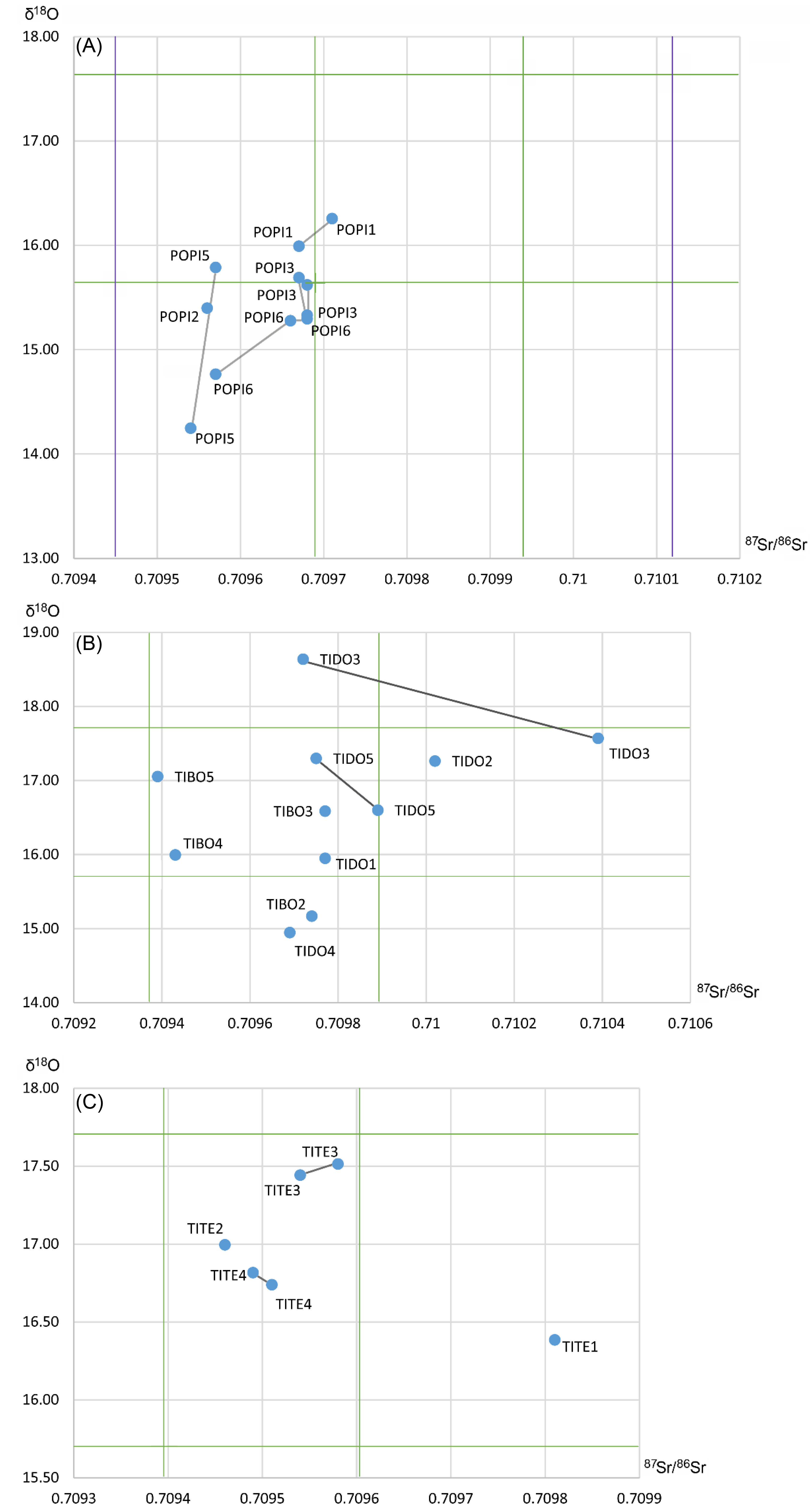

Supplement: S20 Fig — (A) At the Polgár-Piócási-Dűlő (POPI) site. The light green lines represent the local oxygen and strontium isotope baseline ranges. The violet lines represent the micro-regional strontium isotope baseline range. (B) At the Tiszabura Bonishat (TIBO) and Tiszaszőlős-Domaháza-puszta, Réti-dűlő (TIDO) sites. The light green lines represent the local oxygen and strontium isotope baseline ranges. (C) Tiszaföldvár Téglagyár (TITE) site. The green lines represent the site-specific/local oxygen and strontium isotope baseline ranges. (TIF) [file pone.0242745.s023.tif]

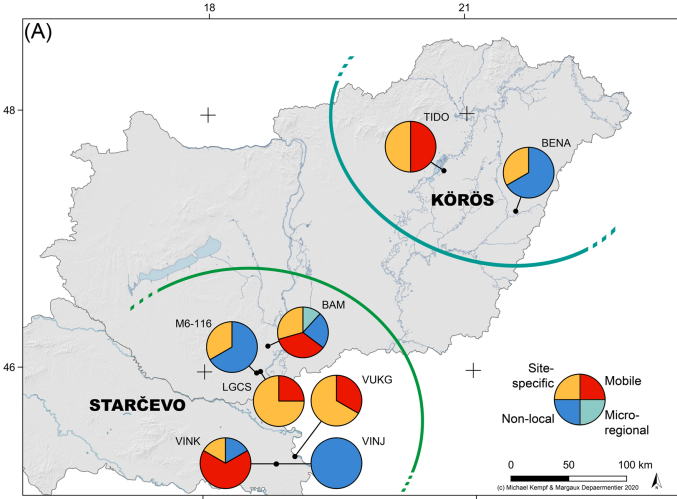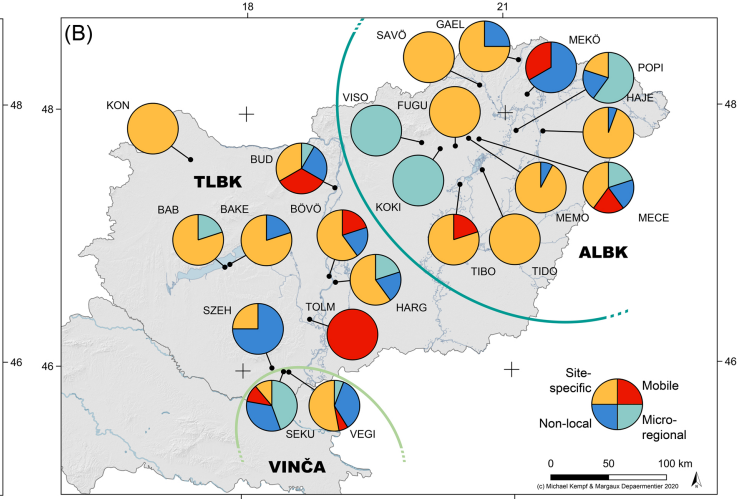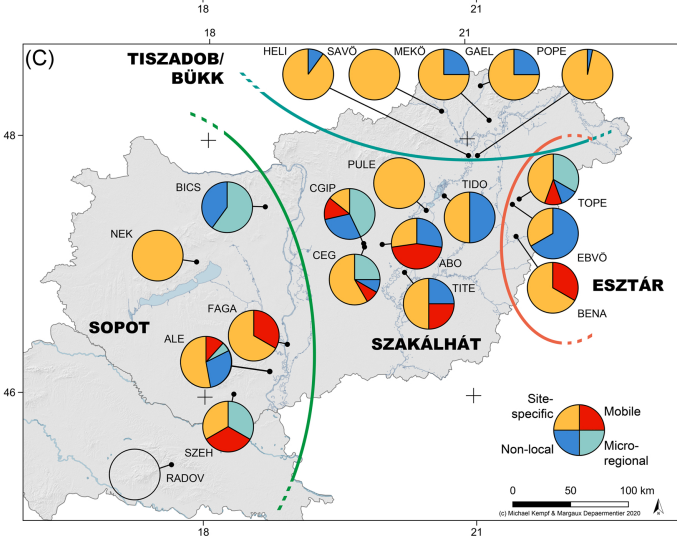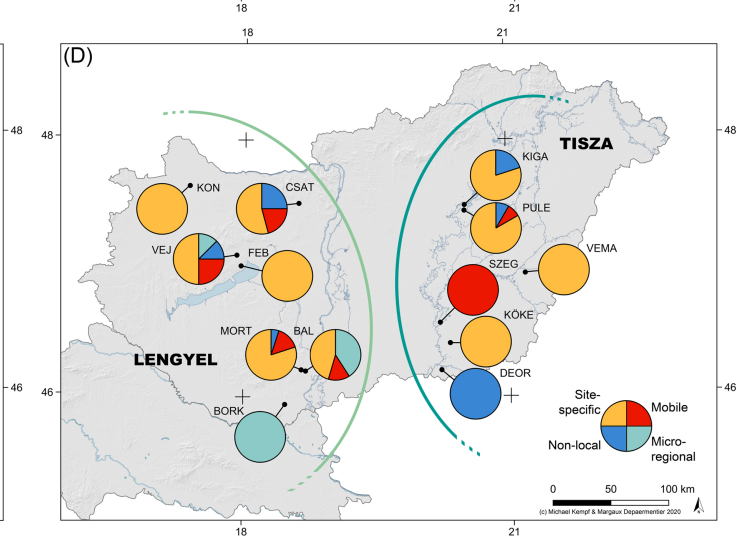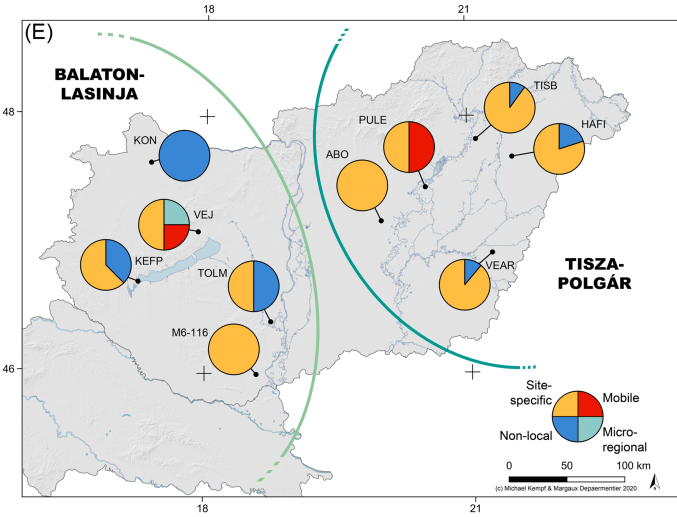

Supplement: S21 Fig — (A) Starčevo and Körös. (B) TLBK, ALBK, and Vinča. (C) Sopot, Esztár, Tiszadob/Bükk, and Szakálhát. (D) Lengyel and Tisza. (E) Balaton-Lasinja and Tiszapolgár. (PDF) [file pone.0242745.s024.pdf]
